# Hypocretin neuron-specific transcriptome profiling identifies the sleep modulator Kcnh4a

**DOI:** 10.7554/eLife.08638

**Published:** 2015-10-01

**Authors:** Laura Yelin-Bekerman, Idan Elbaz, Alex Diber, Dvir Dahary, Liron Gibbs-Bar, Shahar Alon, Tali Lerer-Goldshtein, Lior Appelbaum

**Affiliations:** 1 The Mina and Everard Goodman Faculty of Life Sciences, Bar-Ilan University, Ramat-Gan, Israel; 2 The Leslie and Susan Gonda Multidisciplinary Brain Research Center, Bar-Ilan University, Ramat-Gan, Israel; 3 Toldot Genetics Ltd., Hod Hasharon, Israel; 4 The Weizmann Institute of Science, Rehovot, Israel; 5 Media Lab, Massachusetts Institute of Technology, Cambridge, United states; University of Texas Southwestern Medical Center, United States

**Keywords:** hypocretin, orexin, sleep, transcriptome, kcnh4a, Zebrafish

## Abstract

Sleep has been conserved throughout evolution; however, the molecular and neuronal mechanisms of sleep are largely unknown. The hypothalamic hypocretin/orexin (Hcrt) neurons regulate sleep\wake states, feeding, stress, and reward. To elucidate the mechanism that enables these various functions and to identify sleep regulators, we combined fluorescence cell sorting and RNA-seq in *hcrt:EGFP* zebrafish. Dozens of Hcrt-neuron–specific transcripts were identified and comprehensive high-resolution imaging revealed gene-specific localization in all or subsets of Hcrt neurons. Clusters of Hcrt-neuron–specific genes are predicted to be regulated by shared transcription factors. These findings show that Hcrt neurons are heterogeneous and that integrative molecular mechanisms orchestrate their diverse functions. The voltage-gated potassium channel Kcnh4a, which is expressed in all Hcrt neurons, was silenced by the CRISPR-mediated gene inactivation system. The mutant *kcnh4a (kcnh4a*^-/-^) larvae showed reduced sleep time and consolidation, specifically during the night, suggesting that Kcnh4a regulates sleep.

**DOI:** http://dx.doi.org/10.7554/eLife.08638.001

## eLife digest

Sleep appears to be essential for all animals. The loss of a type of brain cell called the Hypocretin/Orexin (Hcrt) neurons causes the sleep disorder narcolepsy, which disturbs sleep patterns. These neurons also control several other fundamental behaviors and activities, including eating and processing rewards, but it is not clear how Hcrt neurons are able to influence multiple behaviors.

The development and activity of a cell depends to a large extent on the genes it expresses. Yelin-Bekerman et al. have now used genetic techniques to identify a set of genes that are specifically expressed in the Hcrt neurons of zebrafish. Some of these genes are expressed in all of the Hcrt neurons, and some are only expressed in certain subsets of them. Computational methods also revealed a set of “transcription factor” proteins that regulate the expression of clusters of these genes.

Yelin-Bekerman et al. focused on a gene called *kcnh4a*, and found that this encodes an ion channel protein that allows potassium ions to exit the neurons and stop neuronal activity (this activity is also known as an “action potential”). This gene is expressed in all Hcrt neurons. Further experiments showed that zebrafish that lack the potassium channel sleep less during the night. This therefore suggests that the potassium channel is important for regulating sleep.

Future studies of the genes that are enriched in Hcrt neurons could uncover the mechanisms that enable the neurons to play a role in such a diverse range of processes, including feeding and sleep-wake cycles. These studies should enhance our understanding of the role of sleep and may help to develop treatments for metabolic and sleep disorders.

**DOI:** http://dx.doi.org/10.7554/eLife.08638.002

## Introduction

Sleep is a fundamental behavior that benefits the brain and sleep disorders affect a large portion of the world’s population (Shepard et al., 2005). Thus, it is essential to identify and understand the role of the neuronal circuits and genes that regulate sleep. The hypothalamus centralizes sleep regulation and maintains essential physiological processes, including growth, reproduction, body temperature, stress, reward, feeding, and circadian rhythms (Salin-Pascual et al., 2001; Mignot et al., 2002; Saper et al., 2005; Saper, 2006; Begg and Woods, 2013; Dietrich and Horvath, 2013; Sternson, 2013; Muindi et al., 2014). These functions are mediated by several hypothalamic nuclei that interact with various neuronal networks. Some of these nuclei, such as the suprachiasmatic nucleus (SCN), which is the master circadian oscillator (Welsh et al., 2010), have been well characterized both anatomically and physiologically, while the neuronal identity and function of other nuclei is less understood (Rolls et al., 2010; Sakurai and Mieda, 2011; Dietrich and Horvath, 2012). The hypocretin (Hcrt, also called orexin) neurons secrete the Hcrt neuropeptides and are located in the lateral hypothalamus (LH). These hypothalamic neurons project to wide areas in the brain, including the tuberomammillary nucleus, paraventricular thalamic nucleus, arcuate nucleus, and monoaminergic nuclei (Sakurai, 2007). They were initially implicated in feeding behavior and sleep\wake cycles (de Lecea et al., 1998; Sakurai et al., 1998). Their role in sleep regulation was further strengthened since loss of Hcrt neurons causes the sleep disorder narcolepsy, which is characterized by sleep\wake fragmentation, increased body mass, and cataplexy (loss of muscle tone, often triggered by emotional stimuli) (Chemelli et al., 1999; Lin et al., 1999; Peyron et al., 2000; Schuld et al., 2000; Adamantidis et al., 2007; Burgess et al., 2012). However, extensive research showed that the function of Hcrt neurons is much broader and also includes regulation of energy homeostasis, pain, emotion, stress response, and reward (Sakurai, 2007; Rolls et al., 2010; Boutrel et al., 2013). The Hcrt neurons regulate this variety of brain functions through interactions with peptide-secreting neurons and with the monoaminergic, dopaminergic, and limbic systems, among others (Tsujino and Sakurai, 2013).

How do Hcrt neurons serve as a multifunctional hypothalamic system? Clearly, secretion of the neuropeptide Hcrt is a key pathway. A single *hcrt* gene encodes for the precursor polypeptide prepro-Hcrt, which is cleaved to produce two Hcrt neuropeptides. The actions of the Hcrt neuropeptides are mediated via two Hcrt G-protein–coupled receptors (Hcrtrs) (Sakurai, 2007). In addition, the synaptic release of glutamate from Hcrt neurons has been shown to affect the activity of post-synaptic target neurons (Henny et al., 2010; Schone et al., 2012). However, Hcrt neurons contain additional proteins that are likely involved in mediating their development, plasticity, and diverse functions. To date, only a few Hcrt-neuron–specific genes were substantially characterized and, except for *hcrt*, none of them are exclusively expressed in Hcrt neurons (Meister and Håkansson, 1998; Broberger, 1999; Reti et al., 2002; Blouin et al., 2005; Crocker et al., 2005; Belcher et al., 2006; Florenzano et al., 2006; Appelbaum et al., 2007; Honda et al., 2009; Silva et al., 2009; Appelbaum et al., 2010; Cvetkovic-Lopes et al., 2010; Dalal et al., 2013; Liu et al., 2015). A comprehensive and specific gene-expression profiling of Hcrt neurons will enhance the understanding of Hcrt neuronal networks and its diverse functions.

Three studies have described the gene-expression profile of Hcrt neurons in rodents (Honda et al., 2009; Cvetkovic-Lopes et al., 2010; Dalal et al., 2013). First, RNA array was used to study the effect of loss of Hcrt neurons on the expression of hypothalamic transcripts in Hcrt-neuron–ablated mice (Honda et al., 2009). Later, using affinity purification of RNAs and transgenic mice that express FLAG-tagged poly(A)-binding protein, specifically in Hcrt neurons, polyadenylated mRNA was isolated and classified (Cvetkovic-Lopes et al., 2010). Finally, the translating ribosome affinity purification technique that targets HCRT-producing neurons, was used to isolate Hcrt cell-specific RNA in mice (Dalal et al., 2013). These extensive studies resulted in a list of genes expressed in Hcrt neurons. However, in the opaque mammalian brain, isolation of the entire Hcrt neuron population is challenging because a few thousand Hcrt cells are intermingled with other hypothalamic neurons. In addition, all studies used microarray technology, which limits gene resolution and requires *a priori* knowledge of transcript content.

The zebrafish has become a valuable model for the study of specific neuronal populations in live animals. It is a simple and diurnal vertebrate that combines powerful genetic tools with conserved anatomy and function of the brain (Severi et al., 2014; Thiele et al., 2014; Romano et al., 2015). In the last two decades, behavioral criteria have been used to characterize sleep in zebrafish (Zhdanova et al., 2001; Prober et al., 2006; Yokogawa et al., 2007; Sigurgeirsson et al., 2011; Elbaz et al., 2012). Similar to mammals, the Hcrt neurons are located in the zebrafish hypothalamus but, in contrast to mammals, the zebrafish Hcrt system contains only a few neurons, making it a relatively simple system to study (Kaslin et al., 2004; Faraco et al., 2006). Functional studies using Hcrt-neuron–specific genetic ablation, as well as genetic manipulation of the *hcrt* ligand and receptors, showed that the Hcrt system regulates sleep and wake in zebrafish (Prober et al., 2006; Yokogawa et al., 2007; Elbaz et al., 2012). In addition, the zebrafish Hcrt neurons induce feeding behavior (Yokobori et al., 2011), as is the case in mammals. Recently, in order to study Hcrt-neuron specification, a screen for regulatory factors was conducted in the early stages of zebrafish development [26 hr post-fertilization (hpf), (Liu et al., 2015)]. Similar to mammals (Dalal et al., 2013), microarray gene-expression analysis revealed that the LIM homeobox transcription factor Lhx9, which is widely expressed in the brain, including in the Hcrt neurons, can induce the specification of Hcrt neurons (Liu et al., 2015). In the present work, we used 7-days-post-fertilization (dpf) transgenic zebrafish larvae expressing EGFP under the promoter of *hcrt* (Appelbaum et al., 2009), to identify genes that regulate Hcrt-neuron function. The *hcrt:EGFP* larvae were used to specifically isolate Hcrt neurons by fluorescence-activated cell sorting (FACS). Using whole transcriptome RNA sequencing (RNA-seq), meticulous bioinformatic analysis, and extensive anatomical validations, a novel set of Hcrt-neuron–specific genes was identified. Furthermore, the role of the voltage-gated potassium channel Kcnh4a in regulating sleep architecture was studied.

## Results

### Isolation of Hcrt neurons

**Figure 1.**
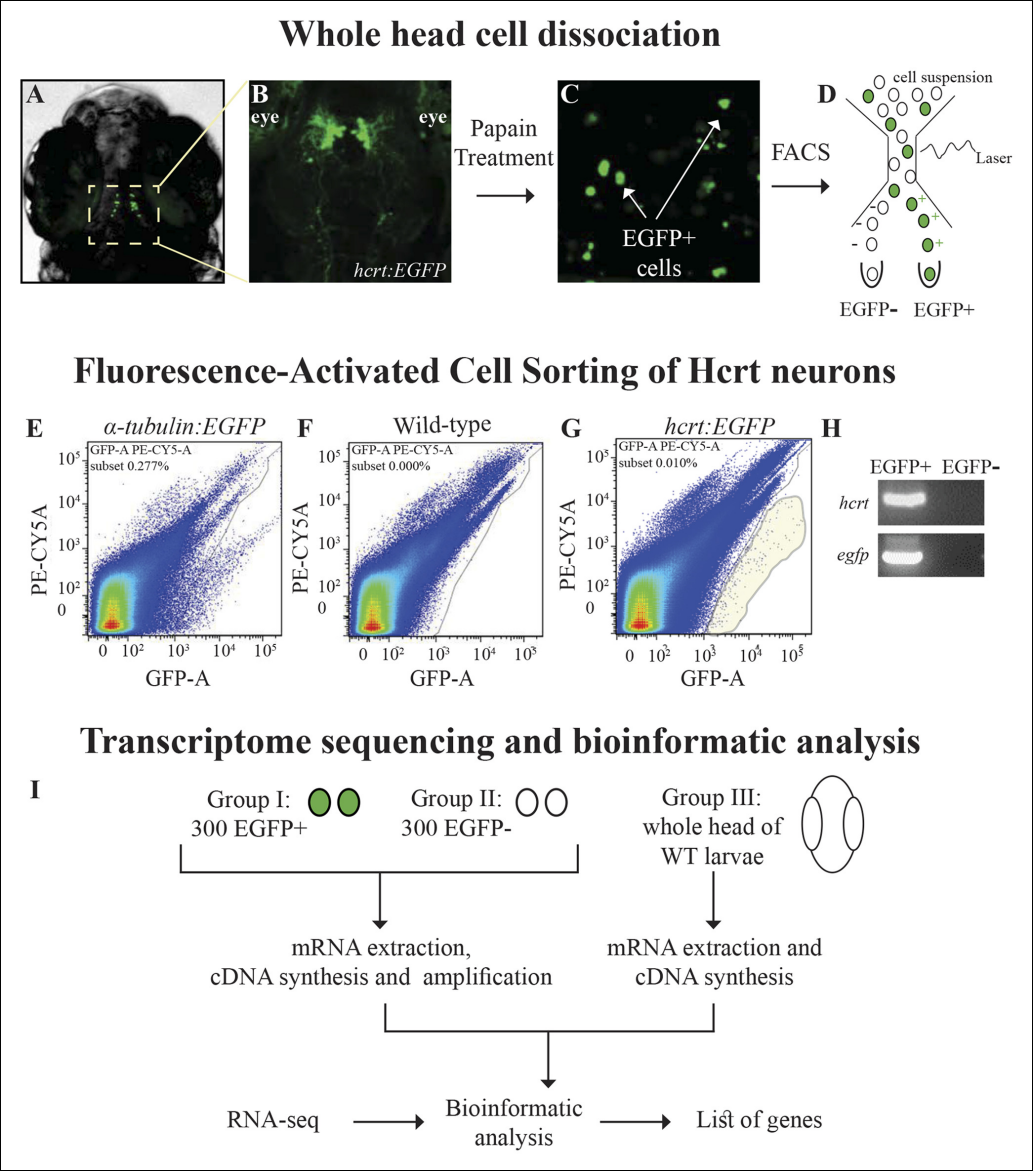
Isolation of Hcrt neurons, RNA-seq, and experimental design. (****A****) Dorsal view of 6 dpf *hcrt:EGFP* larvae. (****B****) Dorsal view of the hypothalamus region of 6 dpf *hcrt:EGFP* larvae expressing EGFP in Hcrt neurons. (****C****) Cell suspension from the whole head of 6 dpf *hcrt:EGFP* larvae. (**D–G**) The cells were sorted based on size and fluorescence intensity. The fluorescence thresholds (gray curve) were set based on larvae expressing EGFP under the control of α-tubulin promoter (positive control) (****E****) and WT larvae (negative control) (****F****). Positive EGFP cells (EGFP^+^) sorted from *hcrt:EGFP* larvae are marked with gray shade (****G****). (****H**)** PCR amplification of hcrt and egfp was performed on cDNA synthesized from EGFP^+^ and EGFP^-^ cells sorted from *hcrt:EGFP* larvae. (****I****) FAC-sorting yielded two groups of cells: Group I containing EGFP^+^ and Group II containing EGFP^-^ cells. A third group contained cells from whole head of WT larvae. The cDNA of groups I and II was amplified and the three groups were then subjected to RNA-seq and bioinformatic analysis to obtain a list of Hcrt-neuron–enriched genes. **DOI:** http://dx.doi.org/10.7554/eLife.08638.003

In order to isolate the Hcrt neurons, the transgenic *hcrt:EGFP* zebrafish (Appelbaum et al., 2009), which enables specific visualization and manipulation of the entire population of Hcrt neurons (16-–20 cells per larva), was used. At 7 dpf, the heads of *hcrt:EGFP* larvae (Figure 1A,B) were dissociated, and EGFP-positive (EGFP^+^) cells from the cell suspension sample (Figure 1C) were sorted by FACS (Figure 1D–G). The sorting thresholds were set to accurately detect the small amounts of cells expressing EGFP while avoiding the auto-fluorescent cells derived primarily from the eyes of the larvae (Figure 1B). In order to calibrate the threshold and additional FACS parameters, *α-tubulin:EGFP*-injected larvae (Figure 1E), which expressed EGFP in the entire central nervous system (CNS), were also FAC-sorted. To avoid off-target sorting of EGFP-negative (EGFP^-^) cells and to set the threshold of EGFP^+^ cells, we applied the same parameters and filters to a cell suspension sample derived from wild-type (WT) larvae (Figure 1F). As expected, the number of EGFP^+^ cells sorted from *hcrt:EGFP* larvae (Figure 1G) was low compared with the number of cells sorted from *α-tubulin:EGFP*-injected larvae. EGFP^+^ cells were not detected in WT larvae (Figure 1F). Using this technique, we collected 300 EGFP^+^ and 300 EGFP^-^ cells from *hcrt:EGFP* larvae in three independent experiments. To verify that the EGFP^+^ cells were Hcrt neurons, RNA extraction was performed, followed by reverse transcription PCR (RT-PCR) assays. While *hcrt* and *egfp* were detected in EGFP^+^ cells, they were not amplified in EGFP^-^ cells (Figure 1H). These results show that the EGFP^+^ cells mostly contain Hcrt neurons, while the EGFP^-^ group contains a heterogeneous population of cells from the whole larva head. Since the amount of RNA extracted from 300 cells was extremely low (below 1 pg/µl) and required amplification before deep sequencing, RNA was extracted from a third control group of cells derived from a whole head of 7 dpf WT larvae. This group helped to distinguish false positive signals that might have resulted from the amplification, and covered genes that were widely expressed in the head and not restricted to 300 EGFP^-^ cells. The RNA of the three groups: EGFP^+^, EGFP^-^, and whole head, was subjected to RNA-Seq and bioinformatic analysis (Figure 1I) to obtain a list of Hcrt-neuron–enriched genes.

### Systematic identification and spatial characterization of genes enriched in Hcrt neurons

**Figure 2.**
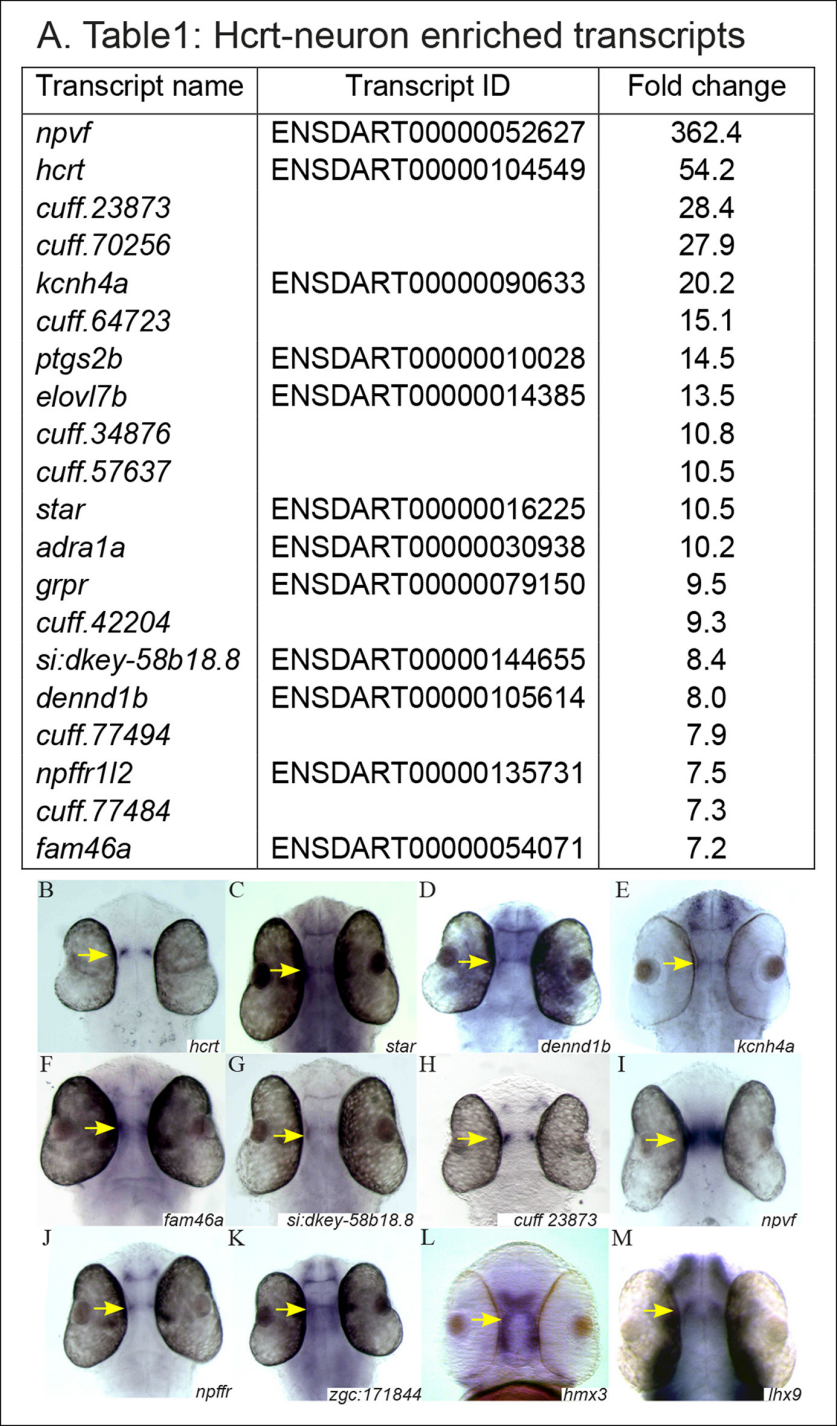
The expression pattern of selected candidate Hcrt-neuron–specific genes. (****A****) Table presenting the top 20 Hcrt-enriched transcripts. (**B–M**) Dorsal view of whole-mount ISH-stained 2 dpf WT larvae. Based on the RNA-seq and the bioinformatic analysis, the expression pattern of selected candidate Hcrt-neuron–specific genes was determined. The expression pattern of *hcrt* (****B****) was used for comparison. **DOI:** http://dx.doi.org/10.7554/eLife.08638.004 10.7554/eLife.08638.005Figure 2—source data 1.Hcrt-neuron enriched transcripts.All the genes detected have *p* <0.01 with Bonferroni correction for multiple testing (Materials and methods).**DOI:** http://dx.doi.org/10.7554/eLife.08638.005

We aimed to identify novel players that regulate the myriad of processes coordinated by Hcrt neurons. Thus, the RNA-seq data from EGFP^+^, EGFP^-^, and whole-head groups (http://www.ncbi.nlm.nih.gov/sra, PRJNA283169) were analyzed *in silico*. Initially, the raw read counts were normalized to transcripts per million (TPM), and a gene was considered to be preferentially expressed in the Hcrt cells only if its normalized expression level was at least 100 TPM in EGFP^+^ cells. In addition, the expression levels were required to be at least 7 times more abundant in the EGFP^+^ than in both controls. These criteria stipulated a high level of specificity to the EGFP^+^ samples relative to the control samples. The bioinformatic analysis identified 20 transcripts that were found to meet these criteria (p<0.01, Figure 2A). Among the 20 transcripts, 12 were annotated genes and 8 were non-annotated transcripts. Notably, the *hcrt* gene was expressed at a level of 300 TPM in EGFP^+^ and below 10 TPM in both control samples. The identification of an *hcrt* gene confirmed the specificity of the cell sorting, the RNA-seq, and the bioinformatic analysis.

**Figure 3.**
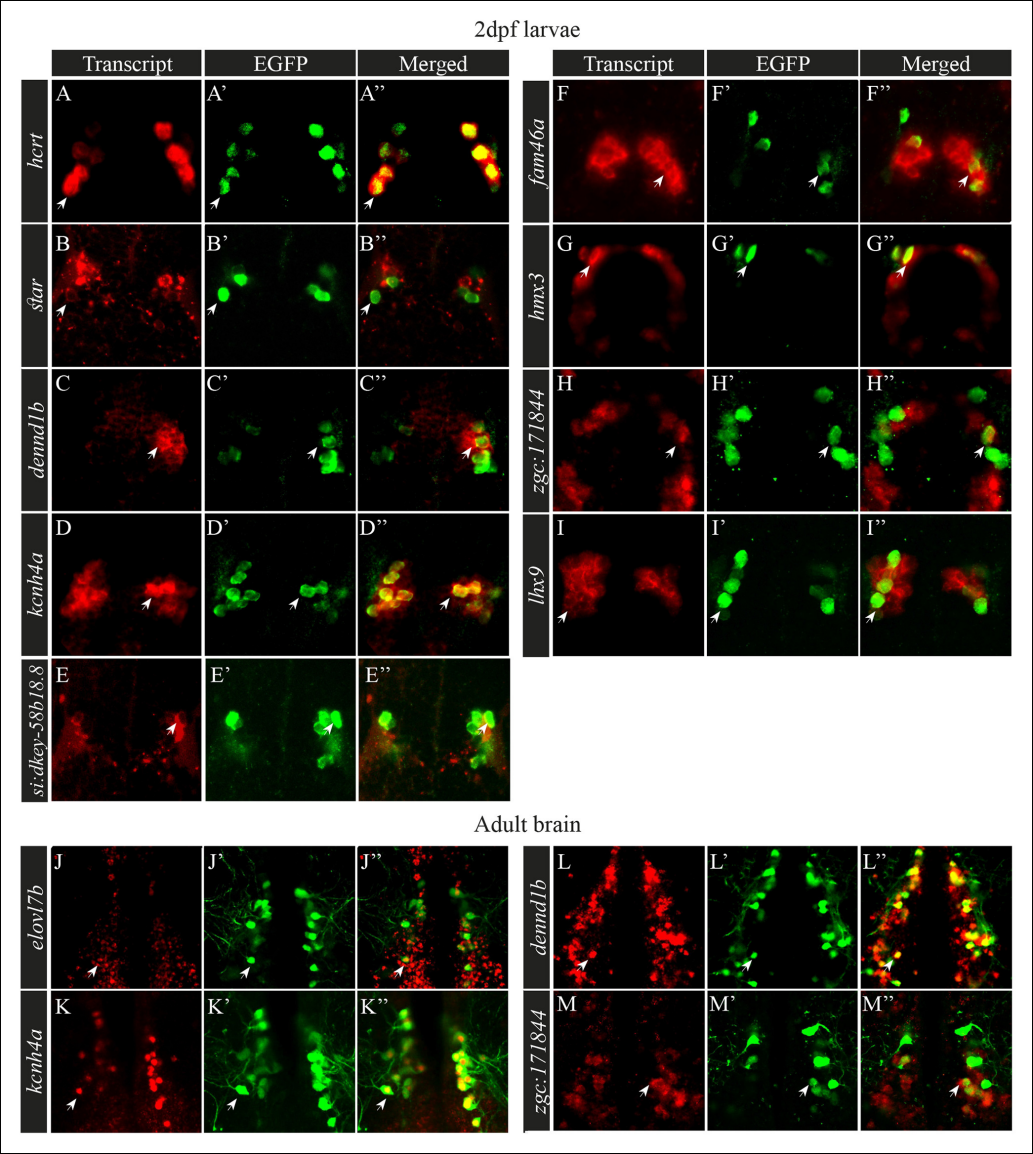
Selected candidate genes are expressed in Hcrt neurons. (**A**–**I''**) Double fluorescent staining of the candidate genes (red) and EGFP (green) was performed in 2 dpf *hcrt:EGFP* larvae using whole-mount ISH and immunofluorescence, respectively. White arrows indicate representative co-expressing cells. All confocal images show single plane view of 0.5 µM width. (**J–M''**) Double fluorescent ISH and immunofluorescence experiments in brain sections of *hcrt:EGFP* adult zebrafish. Co-localization of candidate genes (red) and EGFP (green) in Hcrt neurons is shown. All images show single plane view of 0.5 µM width. **DOI:** http://dx.doi.org/10.7554/eLife.08638.006

In order to validate the bioinformatic results and to determine the spatial expression pattern of the candidate genes, whole-mount *in situ* hybridization (ISH) was performed on 2 dpf WT larvae (Figure 2B–M) using gene-specific probes for the enriched genes (Figure 2A). Nine of them were found to be expressed in the hypothalamic area (*hcrt, star, dennd1b, kcnh4a, fam46a, si:dkey-58b18.8, cuff23873.1, npvf,* and *npffr*; Figure 2B–M). Five transcripts (*adra, ptgs2b, grpr, cuff64723,* and *cuff77494,*) could not be amplified, and the expression of six transcripts (*elovl7b, cuff34876, cuff70256, cuff442204, cuff57637,* and *cuff77484*) was not detected at the 2 dpf larval stage. To test whether these genes were expressed in later developmental stages, their expression was studied in adults. However, only *elovl*7b showed a detectable expression in the hypothalamus (Figure 3J–J"). The hypothalamic expression pattern of the candidate genes was similar to the expression pattern of *hcrt* (Figure 2B), suggesting that the candidate genes may be expressed in Hcrt neurons.

The high percentage of genes that showed hypothalamic expression hints at significant efficiency of the FACS and RNA-seq experiments. Thus, in order to find more Hcrt-neuron–specific genes, we relaxed the bioinformatic parameters to 10 TPM and 3.6 times higher abundance in EGFP^+^ cells than in the control groups. This analysis revealed 212 transcripts that met the criteria (p<0.01, Figure 2—source data 1), among them, 146 were non-annotated (called *cuff*-serial number) and 66 were annotated genes. The functional roles of the annotated genes are diverse and include, for example, regulation of metabolism [such as ELOVL fatty acid elongase 7b (*elovl7*b)], sleep (*lhx9*), synaptogenesis and synaptic plasticity [such as the guanine nucleotide exchange gene (*denndbl*)]. Some of the non-annotated transcripts were likely long, non-coding RNA (lncRNA) since they were longer than 200 bp, did not include a coding sequence, and were located in intergenic regions (Perkel, 2013). lncRNAs regulate transcription and epigenetic processes and may be involved in the regulation of splicing and translation (Mercer et al., 2009). Notably, some non-annotated transcripts were located in the zebrafish genome near Hcrt-enriched genes. In addition to the 8 genes tested (Figure 2C–J), we attempted to examine the expression of selected candidate genes that demonstrate relatively lower enrichment in Hcrt neurons (Figure 2—source data 1). We selected *zgc:171844,* H6 homeobox 3 (*hmx3*), and *lhx9,* which were located in the bottom 100 genes in the list (Figure 2—source data 1). Previous work showed that *lhx9* is expressed in Hcrt neurons in mammals and zebrafish (Dalal et al., 2013; Liu et al., 2015) and that *hmx3* is expressed in Hcrt neurons in the early stages of zebrafish development (Liu et al., 2015). Similar to the genes that demonstrated high fold change (Figure 2A), these three genes were also expressed in the hypothalamus, where *hcrt* is expressed (Figure 2K–M), suggesting that a large portion of the 212 transcripts (Figure 2—source data 1) may be expressed in the Hcrt neurons.

### Identification of genes localized in Hcrt neurons

Single-probe ISH analysis showed that selected candidate transcripts are expressed in the hypothalamus and that their spatial expression pattern is reminiscent of the expression of the *hcrt* gene (Figure 2). To test whether these transcripts are expressed in Hcrt neurons, we performed whole-mount fluorescent ISH using probes for the candidate genes, coupled with immunofluorescence staining using EGFP antibody, in *hcrt:EGFP* 2 dpf larvae and adults. To verify the efficiency and specificity of this assay, co-localization of *hcrt* and EGFP was initially confirmed (Figure 3A–A’'). Double staining showed that among the 11 transcripts tested, 8 transcripts (*star, dennd1b, kcnh4a, fam46a, hmx3, zgc171844, lhx9,* and *si:dkey-58b18.8*) co-localized with EGFP in Hcrt neurons (Figure 3B–I''). While *kcnh4a, hmx3, lhx9* and *dennd1b* were expressed in most Hcrt neurons, *star, fam46a,* and *zgc171844*, were expressed in a subset of the Hcrt neurons. In addition to their expression in Hcrt neurons, these transcripts were also expressed in other brain regions, particularly other hypothalamic areas and the forebrain. In contrast, *si:dkey-58b18.*8 demonstrated relatively weak expression that was predominantly apparent in Hcrt neurons (Figure 3E–E''). Further anatomical analysis in *hcrt:EGFP* adult brain sections was performed on four transcripts (Figure 3J–M''): *elovl7b,* which did not show expression in the earlier developmental stages (Figure 3J–J''), *kcnh4a* (Figure 3K–K''), *dennd1b* (Figure 3L–L''), and *zgc171844* (Figure 3M–M''). Double staining in adults showed that *kcnh4a* and *elovl7b* are detected in all Hcrt neurons, while *dennd1b* is expressed in about half of the Hcrt neurons and *zgc171844* in about a third of the neurons. Notably, the portion of co-localization with EGFP in larvae was similar to that in adults. Altogether, the anatomical results validated the RNA-seq and bioinformatic analysis, which provide a comprehensive list of Hcrt neuron-specific transcripts. The spatial expression of these transcripts in subpopulations of Hcrt neurons indicates that Hcrt neurons are not a uniform population but rather heterogeneous neurons. Understanding the function of these transcripts will provide the basis to elucidate the mechanism that regulates the multifunctions of Hcrt neurons.

### Identification of hypothalamic neuronal populations located adjacent to Hcrt neurons

**Figure 4.**
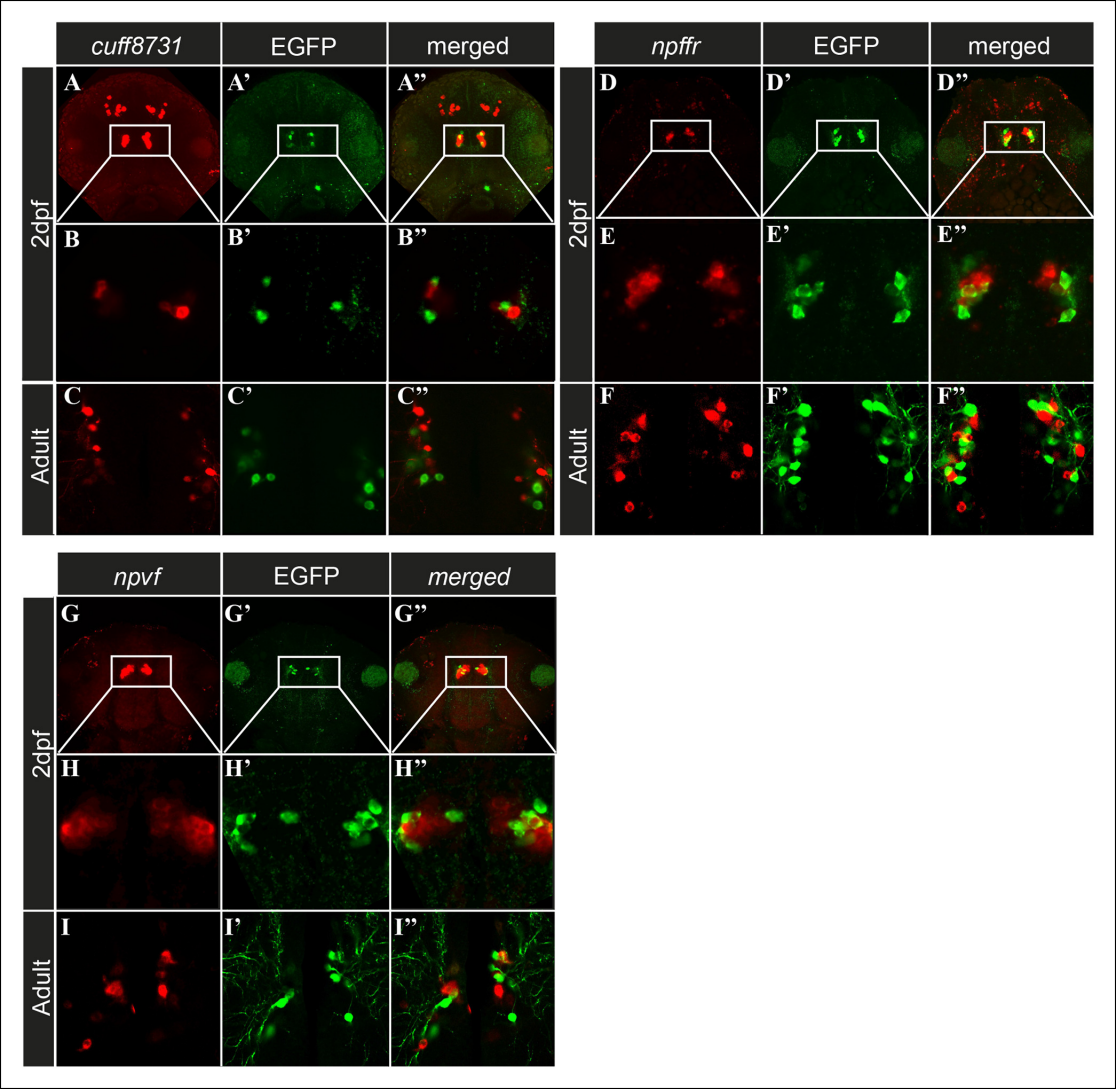
Candidate genes are expressed in cell populations located adjacent to Hcrt neurons. (**A–I''**) Fluorescent ISH and immunofluorescence experiments in 2 dpf larvae and adult *hcrt:EGFP* zebrafish showing three candidate genes that are expressed adjacently to Hcrt neurons within the hypothalamus. (**A–A'', D–D'', G–G''**) Dorsal view of the heads showing the whole expression pattern of the genes in 40 µM z-stack. (**B–B'', E–E'', H–H**) Dorsal view of single 0.5 µM plane in 2 dpf larvae. (**C–C'', F–F'', I–I''**) Dorsal view of single 0.5 µM plane in adult brain section. **DOI:** http://dx.doi.org/10.7554/eLife.08638.007

The histochemical assays revealed transcripts expressed in Hcrt neurons. However, three candidate transcripts (*cuff.23873, npvf,* and *npffr*; Figure 2H–J) labeled distinct hypothalamic populations of neurons that intermingled with Hcrt neurons, but co-localization was not detected (Figure 4). These cell populations were located in the immediate vicinity of the Hcrt neurons in the hypothalamus. While, *cufff.23873* (Figure 4A–C'') and *npffr* (Figure 4D–F'') were also expressed in the forebrain area, *npvf* (Figure 4G–I'') showed a specific hypothalamic expression pattern. The finding of transcripts that were apparently not expressed in Hcrt neurons in the transcriptome, could be due to the adhesion of hypothalamic cells adjacent to Hcrt neurons during the FACS procedure, or because these transcripts are also expressed by Hcrt neurons but below ISH detection levels. Nonetheless, these transcripts are predominantly expressed in hypothalamic neurons and may interact with Hcrt neurons to form neuronal networks that mediate the functions of Hcrt neurons.

### Hcrt-neuron–specific genes are predicted to share similar transcription regulation

**Figure 5.**
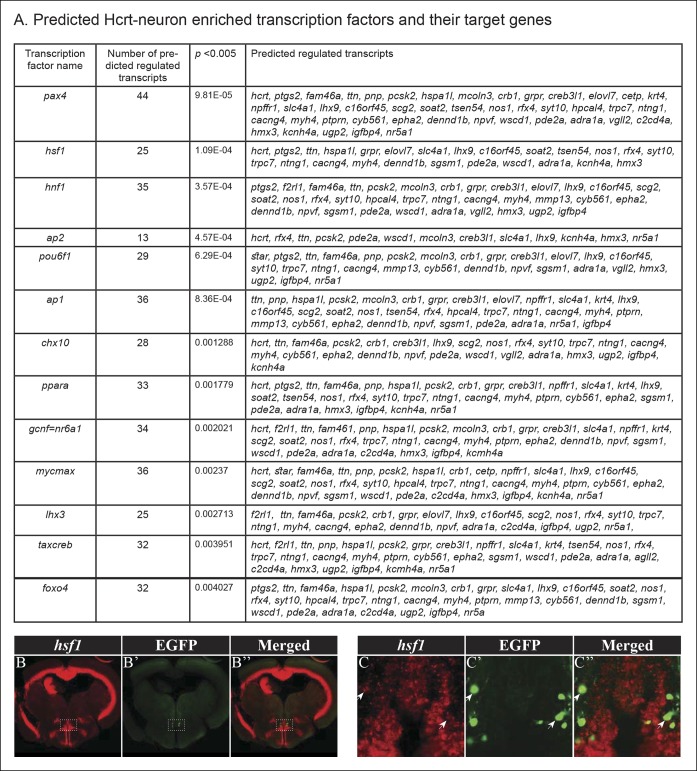
Predicted TFs that regulate the expression of Hcrt-neuron–specific transcripts. (****A****) TFs with a p <0.005 and their target Hcrt-neuron–specific genes. For each of these transcription factors, a combined score for each gene is calculated according to all of the predicted binding sites in its promoter. Therefore, a gene with an overrepresented binding site of a TF will have a high score for that TF and a lower p value. (**B–C''**) Double fluorescent ISH of *hsf1* and immunofluorescence staining of EGFP in *hcrt:EGFP* adult brain section. Single plane (0.5 µM width) view of the Hcrt-neuron region (**C**–**C''**). Arrows mark representative EGFP and *hsf1* co-expressing cell. **DOI:** http://dx.doi.org/10.7554/eLife.08638.008 10.7554/eLife.08638.009Figure 5—source data 1.Predicted Hcrt-neuron enriched transcription factors and their target genes.**DOI:** http://dx.doi.org/10.7554/eLife.08638.009

The mechanism that regulates the specific expression of transcripts in Hcrt neurons and the identity of the transcription factors (TFs) is unclear. To identify candidate TFs that can regulate multiple Hcrt-neuron–specific genes, a map of possible TF binding sites was generated based on the 48 most enriched transcripts. Conserved sequences in the predicted regulatory region of each Hcrt-neuron–specific gene were characterized, and the matched TFs that potentially bind to these sequences were identified. This analysis revealed 68 putative TFs (Figure 5A and Figure 5—source data 1), among them, 13 showed significant enrichment in the top 48 Hcrt-specific transcripts (p<0.005, Figure 5A) including *nr6a1*, which is a regulator of *hcrt* in mice (Tanaka et al., 2010). Notably, this analysis suggests that several specific TFs regulate numerous Hcrt-neuron–specific genes (Figure 5A). For example, the heat shock transcription factor 1 (*hsf1*) is predicted to regulate 25 Hcrt-neuron–enriched genes: *hcrt, ptgs2, ttn, hspa1l, grpr, elovl7b, slc4a1, lhx9, c16orf45, soat2, tsen54, nos1, rfx4, syt10, trpc7, ntng1, cacng4, myh4, dennd1b, sgsm1, pde2a, wscd1, adra1a, kcnh4a*, and *hmx3*. To test whether this predicted TF is expressed in Hcrt neurons, fluorescent ISH, using a probe for *hsf1,* and fluorescent immunostaining using an antibody against EGFP, were performed on the brain section of adult *hcrt:EGFP* zebrafish (Figure 5B–C''). This assay showed wide brain expression of *hsf1* and confirmed that *hsf1* is also expressed in Hcrt neurons. In mammals, *hsf1* is a key activator of stress conditions, and the Hsf1 null mice showed major brain morphological alterations (Santos and Saraiva, 2004). In zebrafish, *hsf1* is essential for recovery from ischemic injury in the brain (Tucker et al., 2011). In addition to *hsf1*, the TF binding-site analysis revealed the enrichment of TFs that regulate metabolic processes (*pax4, hnf1, ppara, lhx3, creb*, and *foxo4*), such as control of the levels of glucagon, insulin, somatostatin, lipids, and glucose (Peyron et al., 2000; Reti et al., 2002; Prober et al., 2006; Plengvidhya et al., 2007; Primo et al., 2008; Reiner et al., 2009). In addition, *ap-2*, a TF that is required for sleep-like behavior in *C. elegans* (Turek et al., 2013), was predicted to regulate the transcription of 13 Hcrt neuron-specific genes (Figure 5A). The identification of mutual TF binding sites in the regulatory sequences of Hcrt-neuron–specific genes suggests that several key TFs regulate the development and function of Hcrt neurons.

### Synteny, cloning, and protein structure of Kcnh4a

**Figure 6.**
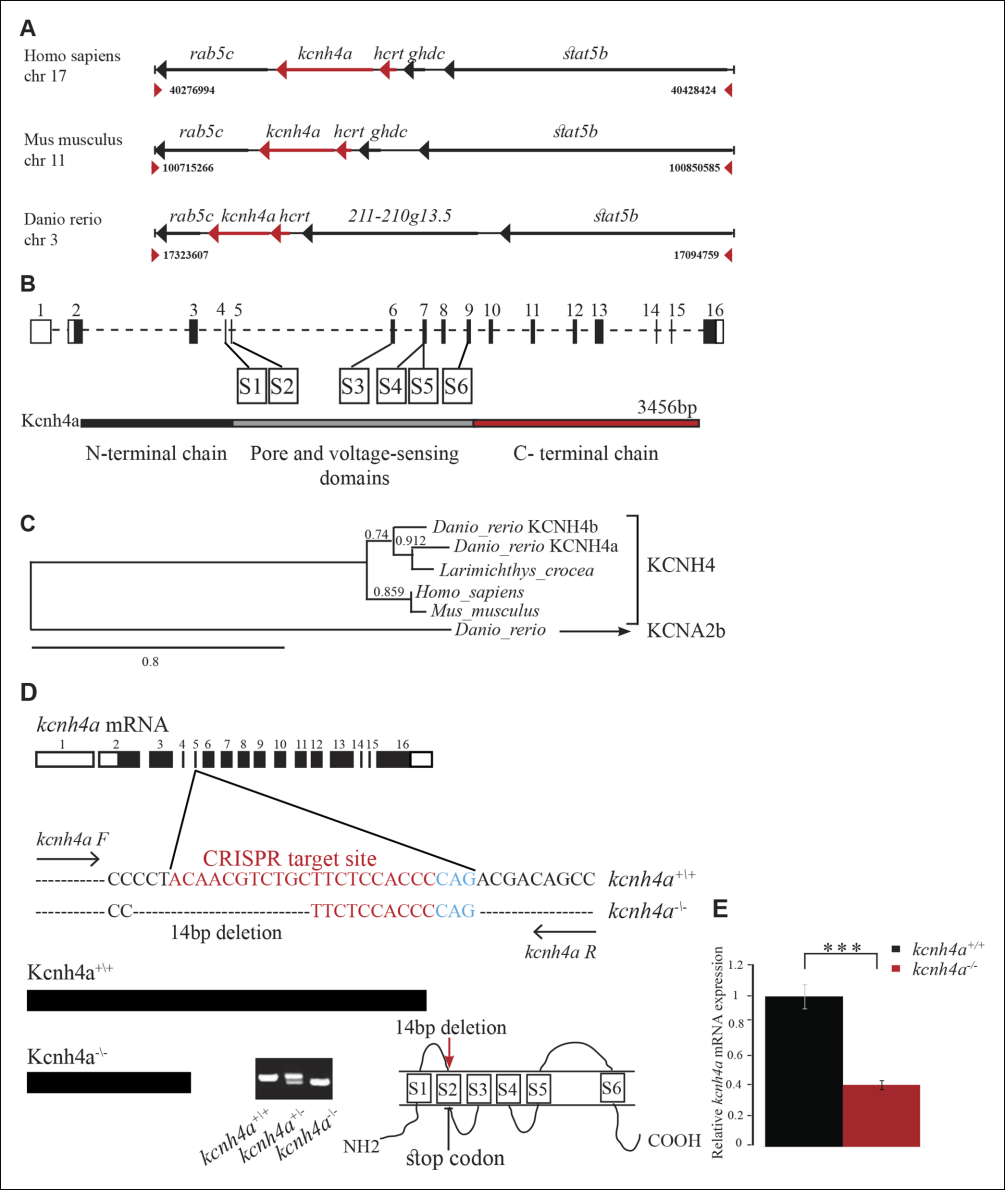
The genomic location, phylogenetic reconstruction and structure of kcnh4a, and the generation of *kcnh4a^-/-^* zebrafish. (****A****) Synteny analysis shows similar genomic context of *hcrt* in zebrafish and mammals. Notably, *kcnh4a* is located a few kbs downstream to *hcrt* in zebrafish and mammals. (****B****) The 16-exon *kcnh4a* gene (black box = exon, white box = UTR) encodes for a voltage-gated potassium channel that includes an N-terminal chain (black bar), pore and voltage-sensing domains (S1-6, grey bar), and the C-terminal chain (red bar). (****C****) A cladogram-style phylogenetic tree depicting the evolutionary conservation of Kcnh4a protein among vertebrates. The tree shows topography as well as distance indicated by the branch support values above corresponding branches. (****D****) Generation of CRISPR-mediated *kcnh4a^-/- ^*zebrafish. A 14 bp deletion was introduced in exon 5 that encodes to the S2 domain. A short mutant allele was visible on agarose gel. (****E****) Quantitative reverse transcription PCR shows reduction of 59% in the expression levels of *kcnh4a* mRNA in *kcnh4a^-/- ^*6 dpf larvae (p <0.001). **DOI:** http://dx.doi.org/10.7554/eLife.08638.010

Among the candidate genes (Figure 2—source data 1), the voltage-gated potassium channel *kcnh4a* was of particular interest because of its genomic location, expression pattern, and predicted role. Two *kcnh4* are present in zebrafish: *kcnh4a* (KR733682) located in chromosome 3 and *kcnh4b* (XM_690738) located in chromosome 12. In contrast to the broad expression of *kcnh4b* (data not shown), *kcnh4a* is expressed specifically in the forebrain and hypothalamus in larvae (Figure 2E). Double ISH and immunofluorescence staining revealed that *kcnh4a* is localized in all Hcrt neurons in both larvae and adults (Figure 3D**–**D'',K–K'') and like Hcrt neurons, hypothalamic *kcnh4a*-expressing neurons are glutamatergic (Figure 6—figure supplement 1). Intriguingly, *kcnh4a* is localized only a few kilobase pairs (kbp) downstream to *hcrt* on the genome, and a synteny analysis showed that the genomic organization of this locus is conserved with mammals (Figure 6A). In humans, *kcnh4a* is located only 3782 bp downstream to the *hcrt* gene, while in zebrafish, the distance between the genes is 5517 bp (Figure 6A). Although *kcnh4a* expression is not restricted to Hcrt neurons, the genomic proximity of the two genes suggests a mutual transcription regulation. Indeed, a significant portion of the TFs predicted to regulate *hcrt* can also bind to *kcnh4a* regulatory sequences (48 out of 52, Figure 5—source data 1).

**Figure 6—figure supplement 1.**
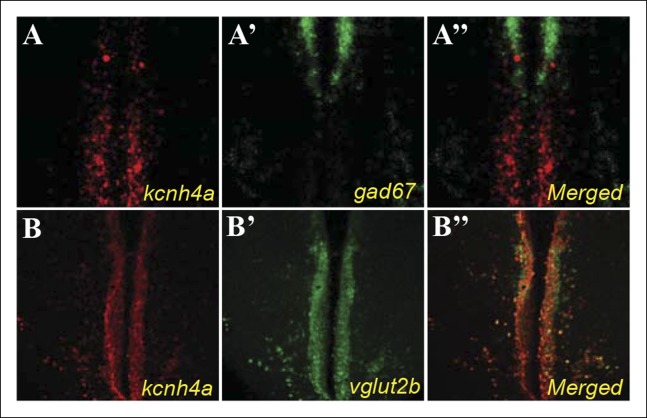
Hypothalamic *kcnh4a*-expressing neurons are glutamatergic. Double in-situ hybridization against *kcnh4a* (red, **A, B**) and *gad67* (green, **A'**), or *vglut2b* (green, **B'**). All pictures are on a single optical plane of 0.5 µm. **DOI:** http://dx.doi.org/10.7554/eLife.08638.011

Vector cloning and sequencing of *kcnh4a* showed that the gene consists of 16 exons, which include the 3456 bp coding sequence (CDS, Figure 6B). The start codon is located within the second exon, preceded by 1884 bp 5’ UTR. Exon 16 includes the stop codon, followed by 1286 bp 3’UTR. Structural bioinformatic analysis of the protein sequence revealed that the Kcnh4a contains the evolutionarily conserved six S domains that characterize the potassium voltage-gated ion channels (Figure 6B) (Choe, 2002). Domains S1**–**S4 constitute the voltage-gated domain that senses changes in membrane potential (Bezanilla, 2000; Swartz, 2008), whereas domains S5**–**S6 form the selectivity pore through which ions can flux (Bezanilla, 2000; Choe, 2002; Swartz, 2008). Next, phylogenetic analysis revealed that the zebrafish Kcnh4a protein is evolutionarily conserved with vertebrate orthologs (Dereeper et al., 2008). As expected, the zebrafish Kcnh4a protein showed the highest homology to the Kcnh4 of another fish (*Larimichthys_crocea*) and, to a lesser extent, to the mammal Kcnh4 proteins (Figure 6C).

### Mild, reduced locomotor activity in *kcnh4a^-/-^*larvae

In order to test the function of Kcnh4a, we established a clustered, regularly interspaced, short palindromic-repeat (CRISPR)-based *kcnh4a* mutant zebrafish (*kcnh4a^-/-^*). A 14 bp deletion mutation was generated in exon 5, which encoded part of the pore domain. This deletion introduced a premature stop codon and is predicted to result in truncated protein (Figure 6D). Furthermore, quantitative reverse transcription PCR (qRT-PCR) showed a reduction of 59% of *kcnh4a* mRNA levels in *kcnh4a ^-/-^* compared to WT-sibling (*kcnh4a^+/+^*) 6dpf larvae (p<0.001, Figure 6E). The founder (F0) fish was outcrossed with WT fish, and experiments were performed on the progeny of inter-crosses between F4 heterozygous fish (*kcnh4a*^*+*/-^).

**Figure 7.**
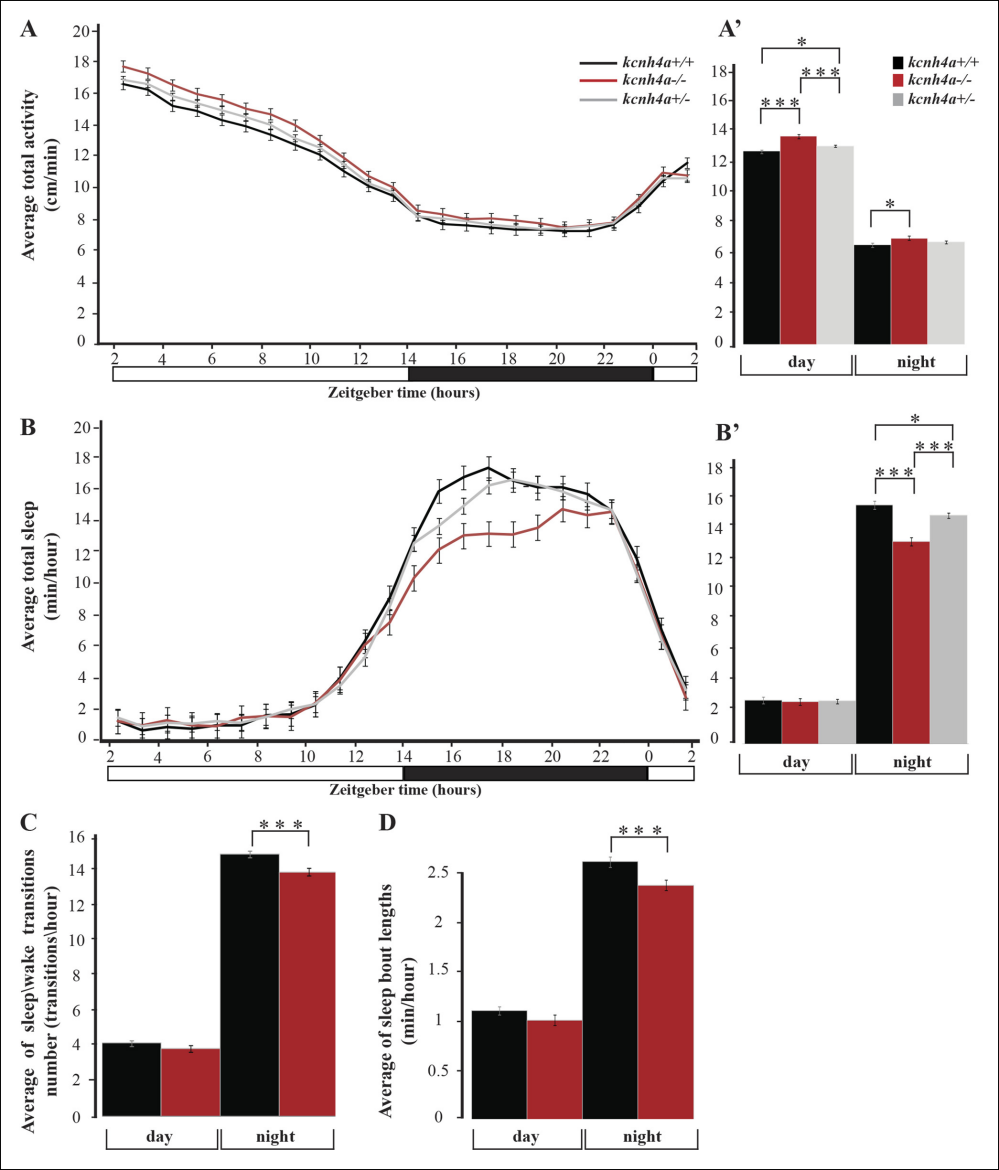
Sleep time and quality are reduced in *kcnh4a*^-/-^ larvae during the night. (****A****) The locomotor activity of *kcnh4a^-/-^* (n=85), *kcnh4a^+^*^/-^ (n=208), and *kcnh4a^+/+^* (n=98) is shown. *kcnh4a^-/-^* larvae exhibit increased locomotor activity compared with *kcnh4a^+/-^* and *kcnh4a /* under LD conditions. (****B****) *kcnh4a^-/-^* larvae showed a significant reduction in sleep time compared with *kcnh4a^+/-^* and *kcnh4a^+/+^* during the night. Bar charts represent the average total locomotor activity (**A'**) and sleep time (**B'**) for each genotype. Values are represented as means ± SEM. (**C,D**) The number of sleep\wake transitions (****C****) and the length of sleep bout (****D****) are decreased in *kcnh4a*-/- larvae during the night. Recording of locomotor activity and sleep was performed in 6 dpf larvae continuously during 24 hr under a 14 hr light/10 hr dark cycle (white and black bars represent light and dark periods, respectively, *p<0.05, **p<0.01, ***p<0.0001, with repeated measures of ANOVA). **DOI:** http://dx.doi.org/10.7554/eLife.08638.012

To study the rhythmic locomotor activity of *kcnh4a^-/-^* zebrafish, high-throughput video-tracking systems were used (Elbaz et al., 2012). The locomotor activity of *kcnh4a^-/-^*(n=85), *kcnh4a^+/-^* (n=209), and *kcnh4a^+/+ ^*(n=98) was monitored during day and night (14 hr light/10 hr dark). As expected, larvae from all three genotypes exhibited rhythmic locomotor activity that peaked during the day (F_[2,180]_ = 14.98; p<0.0001, mixed-effect model with repeated measures; Figure 7A-A’). Notably, *kcnh4a^-/-^*larvae were slightly hyperactive (average: 13.84 ± 0.11) compared to *kcnh4a*^*+*/-^ (average: 13.29 ± 0.07, *t* = -4.19, *df* = 180, p<0.01) and *kcnh4a^+/+^*sibling larvae during the day (average: 12.98 ± 0.1, *t* = 5.73, *df* = 180, p<0.0001). During the night, the differences in locomotor activity were even lower, and the *kcnh4a^-/-^*larvae were slightly more active (average: 8.05 ± 0.13) than the *kcnh4a^+/+^* larvae (average: 7.64 ± 0.12, *t* = 2.28, *df* = 180, p*<*0.05; Figure 7A-A’). These results show that the loss of Kcnh4a mildly increases larval locomotor activity, particularly during the day.

**Figure 7—figure supplement 1.**
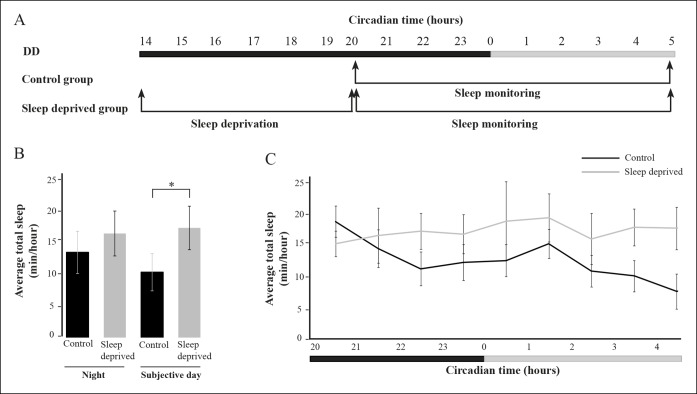
Sleep time is increased following sleep deprivation (SD). (****A****) At 6dpf, larvae were sleep deprived for 6 hr during the night under constant dark conditions (DD) and sleep time was monitored in the following nine hours. (**B,C**) Sleep was recovered in sleep-deprived larvae. Statistical comparisons were performed using Student’s t-tests (*p<0.05). Dark and gray horizontal bars represent night and subjective day, respectively. **DOI:** http://dx.doi.org/10.7554/eLife.08638.013

### Reduced sleep time and altered sleep architecture in *kcnh4a^-/- ^*larvae during the night

Similar to humans, the zebrafish is a diurnal vertebrate that sleeps during the night (Zhdanova, 2011; Elbaz et al., 2013). Using well-established behavioral criteria, sleep in larvae was defined as a period of one or more minutes of immobility, which is associated with an increase in arousal threshold (Prober et al., 2006; Elbaz et al., 2012). A previous study has shown that six hours of sleep deprivation (SD) during the night reduced locomotor activity in the following day (Zhdanova et al., 2001). Similarly, six hours of SD during the night increased sleep time during the following day in 6 dpf larvae (Figure 7—figure supplement 1). Thus, similar to mammals, sleep in zebrafish larvae is regulated by circadian and homeostatic processes.

Voltage-gated potassium channels are activated by membrane depolarization and contribute to neuronal repolarization and repetitive firing (Choe, 2002). Considering this role and the expression of *kcnh4a* in all Hcrt neurons, we tested whether it regulates sleep and wake. Similar to humans, zebrafish are diurnal animals; thus, all three genotypes (*kcnh4a^-/-^,kcnh4a^+/-^*, and *kcnh4a^+/+^*) slept more during the night than during the day (F_[2,180]_ = 14.52; p<0.0001, mixed-effect model with repeated measures, Figure 7B). Remarkably, while the amount of sleep was similar in all genotypes during the day (average: *kcnh4a^-/-^* = 2.76 ± 0.22; *kcnh4a^+/-^* = 2.80 ± 0.14; and *kcnh4a^+/+^* = 2.87 ± 0.21), sleep time was reduced in *kcnh4a^-/- ^*larvae compared with *kcnh4a^+/-^*and *kcnh4a^+/+^* larvae during the night (average: *kcnh4a^-/-^* = 13.08 ± 0.27; *kcnh4a^+/-^* = 14.78 ± 0.17; and *kcnh4a^+/+^* = 15.46 ± 0.25, t = -6.55, *df* = 180, p<0.0001, Figure 7B, B'). In order to examine the sleep architecture, we quantified the number of sleep\wake transitions and the length of sleep bouts. While the number of sleep\wake transitions did not change during the day (*kcnh4a^-/-^* = 3.52 ± 0.16; *kcnh4a^+/+^* = 3.79 ± 0.15), their number was decreased in *kcnh4a^-/-^*larvae during the night (average: *kcnh4a^-/-^* = 12.68 ± 0.19; *kcnh4a^+/+^* = 13.62 ± 0.18, transitions/hr, F_[1,80]_ = 13.16; p<0.0005; *df* = 80, p<0.0005, Figure 7C). In addition, the *kcnh4a^-/-^*larvae exhibit shorter sleep-bout length specifically during the night (average: *kcnh4a^-/-^* = 2.21 ± 0.05; *kcnh4a^+/+^* = 2.43 ± 0.05; minhour *df* = 81, p <0.005, Figure 7D). Thus, the reduction in the number and length of sleep episodes causes global reduction in sleep time during the night in *kcnh4a^-/-^*larvae. These results show that the loss of Kcnh4a affects sleep time and sleep consolidation, specifically during the night. It also suggests that Kcnh4a regulates sleep by repolarization of the membrane potential in sleep-regulating neurons.

## Discussion

How the hypothalamic Hcrt neurons regulate diverse and fundamental physiological functions and what is the molecular mechanism that controls sleep are largely open questions. We revealed the molecular profile of the Hcrt neurons and functionally demonstrated the role of Kcnh4a in regulating sleep. Using FAC-sorting of the whole Hcrt neuronal population and RNA-seq of minute amounts of RNA, 212 Hcrt-neuron–specific transcripts were identified. Combination of fluorescent ISH and immunofluorescence assays confirmed that several transcripts are expressed in Hcrt neurons. The high efficiency and specificity of these anatomical experiments suggest that a large portion of the candidate genes (Figure 2—source data 1) are expressed in Hcrt neurons. Indeed, *lhx9* and *hmx3*, which were ranked lower in the list of candidate genes, were previously shown to be expressed in the early stages of Hcrt-neuron development (Dalal et al., 2013; Liu et al., 2015), and we confirmed these observations in 7 dpf larvae. Thus, these results provide a comprehensive list of genes that are likely to mediate the multifunctions of Hcrt neurons. Comparison between the Hcrt-neuron–specific candidate genes isolated in zebrafish and mice (Dalal et al., 2013) showed that eight genes (*rfx4, lhx9, scg2, vgll2, ptprn, creb3l1, sgsm1,* and *fam46a*) are found in both vertebrates. This genetic similarity is reasonable; however, performing similar cell isolation technique and bioinformatic analysis in both species would have likely increased the list of shared Hcrt-neuron–specific genes. Accumulated data on mammals and zebrafish suggest that the Hcrt neurons are not a homogenous population (Appelbaum et al., 2007; Dalal et al., 2013). Indeed, our co-localization studies showed a diversity of spatial gene expression in Hcrt neurons, varying from partial to complete overlapping with *hcrt*. Thus, the molecular signature suggests that these neurons are divided into subpopulations that may cope with the wide variety of functions of Hcrt neurons. The development and diverse functions of Hcrt neuron subpopulations are predicted to be regulated by Hcrt-neuron–expressed TFs, which target an ensemble of Hcrt-neuron–specific genes.

The role of the isolated Hcrt-neuron–specific genes is diverse. Large arrays of genes are involved in the regulation of metabolism, endocrine systems, synaptic function, neurogenesis, reward, wake, and sleep (Figure 2—source data 1). These functions are correlated with the diverse role of Hcrt neurons. A group of genes includes metabolic and endocrine genes, such as the protein tyrosine phosphatase receptor (*ptprn*), which is implicated in insulin regulation (Saito, 1993; Primo et al., 2008), and the steroidogenic acute regulatory protein (*star*), which regulates the production of steroid hormones from cholesterol in the mitochondria (Stocco et al., 2005; Ings and van der Kraak, 2006). Another gene involved in metabolism is *elovl7*b, which regulates fatty acid metabolism and energy homeostasis. This gene has been linked to lipodystrophy, obesity, and other metabolic disturbances (Brolinson et al., 2008; Chen et al., 2013). These metabolic genes are likely part of the mechanism that regulates feeding and obesity. Thus, in addition to *hcrt*, an imbalance of the fatty acid and glucose-regulating genes and pathways may contribute to the metabolism-related symptoms of narcoleptic patients.

An array of Hcrt-neuron–specific genes are involved in neurogenesis and synaptic plasticity. For instance, the synaptic vesicle protein synaptotagmin X (*syt10),* which is involved in vesicular trafficking and Ca(2 )-dependent exocytosis (Zhao et al., 2003; Craxton, 2010). In addition, the voltage-dependent calcium-channel (*cacgn4*) gene regulates the biophysical properties of α-amino-3-hydroxy-5-methyl-4-isoxazolepropionic acid (AMPA) receptors (Sager et al., 2009) and secretogranin II (*scg2)* encodes to a secretory protein and mediates the packaging and sorting of neuropeptides into secretory vesicles (Fischer-Colbrie et al., 2005; Kassahn et al., 2009). Additional examples include the *denndbl*, which is involved in axon guidance, synaptic plasticity, and synaptic vesicle exocytosis (Wu et al., 2011), and netrin G1 (*ntng1*), which is part of the mechanism that regulates axon guidance during development (Nakashiba et al., 2000; Seo et al., 2009). Altogether, these genes are likely to play a key role in the mechanism that regulates neuritic processes, synaptic plasticity and activity in Hcrt neurons.

In addition to annotated genes, the RNA profiling also revealed a set of long, non-coding RNA (lncRNAs) enriched in Hcrt neurons. lncRNAs regulate gene transcription and expression via various molecular mechanisms. Several studies show that lncRNAs regulate the expression of protein-coding genes, with their genomic loci adjacent to the locus of the specific lncRNA (Goodrich and Kugel, 2006; Mercer et al., 2009). Supporting these observations, among the 16 lncRNAs that were enriched in Hcrt neurons, several were located in the genome next to the Hcrt-neuron–specific genes. For instance, the lncRNA *cuff23873* is placed between the genes *hacl1* and *ankrd28* while *si:dkey-58b18.8* is located in the intergenic region between *pim2* and *rpp40*. Thus, these results suggest that Hcrt-neuron–specific lncRNAs regulate transcriptional and post-transcriptional processes of Hcrt-neuron–specific genes.

The identification of hundreds of Hcrt-neuron–specific candidate genes enabled us to predict the TFs that regulate the expression of these genes. We found a significant enrichment of TFs, which regulate metabolism, sleep, and other physiological processes (Figure 5). For example, the hepatic nuclear factor 1 homeobox (*hnf1*) regulates the expression of genes involved in lipid and glucose transport (Reiner et al., 2009). In the Hcrt neurons, it was expected to regulate 35 out of the 48 Hcrt-neuron–specific genes (Figure 5), including five metabolic genes (*soat2, f2rl1, scg2, grpr*, and *elovl7*b). Another key TF is the peroxisome proliferator-activated receptor alpha (*ppara*), which plays a role in lipid metabolism and satiety (Fu et al., 2003; Chakravarthy et al., 2009). In the Hcrt neurons, this TF is expected to regulate the transcription of 33 Hcrt-neuron–specific genes, such as *ptprn, soat2, f2rl1, scg2*, and *grpr*, which are associated with the balancing of metabolism. Notably, the TF *ap1*, which is associated with sleep induction (Turek et al., 2013), is expected to be a regulator of 13 Hcrt-neuron–specific genes, including *lhx9*, which regulates sleep (Dalal et al., 2013). Intriguingly, *ap1* can also regulate the expression of Hcrt-neuron–specific synaptic genes, such as *cacgn4, nos1*, and *dennd1b*. Since sleep regulates synaptic plasticity in Hcrt axons (Appelbaum et al., 2010), *ap1* might mediate the molecular mechanism that links sleep with synaptic plasticity in Hcrt neurons.

The different players that are expressed in Hcrt neurons modulate the diverse roles of the neurons; however, these functions are also mediated by other hypothalamic neuronal networks. Aside from the Hcrt-neuron–specific genes, we identified three transcripts, *cuff8731, npvf,* and *npffr1*, which are expressed in cells located adjacent to Hcrt neurons. The neuropeptide VF precursor (*npvf*) and its receptor (*npffr1*) regulate nociception, anxiety, learning, and memory (Liu et al., 2001). The NPVF/NPFFR1 system also controls pain and analgesia through interactions with the opioid system (Liu et al., 2001). The opioid system is formed, among others, by Nociceptin that forms the nociceptin/orphanin FQ (N/OFQ) system. This system makes synaptic contacts with Hcrt neurons, inhibiting their activity via pre- and post-synaptic mechanisms. The nociceptin/orphanin FQ (N/OFQ) system also exerts diverse actions in the hypothalamic–pituitary–adrenal (HPA) axis, and is implicated in the neurobiological control of stress and associated adaptive behaviors (Delaney et al., 2012). More specifically, Hcrt neurons are essential in the generation of stress-induced analgesia (SIA), and N/OFQ blocks SIA via inhibition of Hcrt neuron activity (Xie et al., 2008). Altogether, NPVF/NPFFR and Hcrt neurons may interact in the hypothalamus to regulate morphine- and stress-induced analgesia.

Among the candidate Hcrt-neuron–specific genes, we studied the role of *kcnh4a,* which is located adjacent to *hcrt* in the genome and is expressed in all Hcrt neurons. We found that sleep time, sleep\wake transitions, and sleep-bout length are decreased in *kcnh4a^-/-^*larvae during the night. Since potassiumvoltage-gated channels repolarize the cell membrane (Choe, 2002; Bezanilla, 2000; Swartz, 2008), loss of *kcnh4a* may reduce potassium efflux and induce repetitive hyperpolarization, and, ultimately, nighttime wakefulness. Supporting this role, the ether-a-go-go-gene–related (ERG) potassium channel blockers selectively increased waking activity at night in zebrafish (Rihel et al., 2010). The importance of potassium channels for sleep regulation has also been demonstrated in flies. Genetic screen of fly mutants revealed the short sleeper *shaker* mutant. The *shaker* gene encodes a voltage-dependent potassium channel and regulates sleep need and efficiency (Cirelli et al., 2005). Notably, loss of the *shaker* and *kcnh4a* potassium channels similarly affects nighttime sleep, while daytime sleep is unaffected in *kcnh4a^-/-^*larvae. In mice, loss of the voltage-dependent potassium channel Kcna2 decreases non-rapid-eye-movement (NREM) sleep and increases wakefulness (Douglas et al., 2007). These findings suggest that sleep is regulated by neuronal-circuit–specific potassium channels in flies, zebrafish, and mammals. According to this model, in zebrafish, the presence of Kcnh4a in the excitatory Hcrt neurons suggests that Kcnh4a regulates their activity and, ultimately, sleep and wake. In *kcnh4a^-/-^*larvae, the absence of Kcnh4a may cause hyperexcitability of the Hcrt neurons that induces the activity of downstream arousal-promoting targets, such as the paraventricular thalamic nucleus (Huang et al., 2006) and the locus coeruleus (Carter et al., 2010). However, since the expression of Kcnh4a is not restricted to Hcrt neurons and its effect on firing rates in specific neuronal population is not clear, further neurophysiological studies are required to link Kcnh4a, neuronal activity, sleep and wakefulness.

The Hcrt transcriptome identified Kcnh4a as a sleep regulator and provides a platform for future studies on the molecular mechanism that regulates Hcrt-neuron–dependent physiological processes, such as feeding and sleep-wake cycles. In addition, it may also help to identify Hcrt-neuron–specific antigens that trigger the autoimmune response, leading to the specific elimination of Hcrt neurons in narcolepsy (Mahlios et al., 2013). Since the transparent zebrafish offer a wide array of tools to manipulate genes and visualize neuronal-circuit activity in live animals, a future combination of CRISPR-mediated mutants, genetically encoded Ca^2+^ indicators, and optogenetic tools are expected to elucidate the functional role of the Hcrt-neuron–specific genes in a neuronal-circuit–specific manner. These experiments will facilitate our understanding of the mechanism controlling the multifunctional Hcrt neurons.

## Materials and methods

### Fish maintenance

The *hcrt:EGFP, kcnh4a^-/-^*, *kcnh4a^+/-^, kcnh4a^+/+^*, and WT fish were kept in a fish facility under a 14 hr light/10 hr dark cycle (LD) at 28°C (Elbaz et al., 2012) under optimal maintenance conditions, in accordance with the animal protocol approved by the Bar-Ilan University Bioethics Committee. Larvae were generated by paired mating, and raised in incubators and larvae water systems (Elbaz et al., 2012) under LD.

### FAC-sorting

The heads of 7 dpf *hcrt:*EGFP, *α-tubulin:EGFP*-injected, and WT larvae (60 larvae per group) were collected in a 2 ml tube. Cells were then dissociated using the Papain Dissociation System (Worthington Biochemical Corporation, Lakewood, NJ) according to the manufacturer’s protocol. The cells were filtered with a 70 μm cell strainer (BD Transduction Laboratories, San Jose, CA) and washed twice with cold phosphate-buffered saline (PBS). High-speed FACS was performed using an LSRII FACS machine (BD, Bioscience, San Jose, CA). A two-gate FACS technique was used to select only EGFP^+^ cells from non-fluorescent and auto-fluorescent cells. The EGFP^+^ cells were differentiated using SSC-A and FSC-A strategies. As a control, two additional groups of cells were sorted: the first group contained only EGFP^+^ cells derived from the heads of larvae expressing *α-tubulin*:EGFP, and the second group contained only EGFP^-^ cells derived from WT larvae. Then, to separate the EFGP^+^ cells from the EGFP^-^ cells, PE-Cy5-A and GFP-A filters were applied. The cells were collected into a 96-well sterile plate filled with the first RNA purification buffer from the RNeasy Mini Kit (Qiagen, Redwood City, CA). The samples were stored at -80°C until the RNA was purified. Three independent FACS experiments were performed, yielding three samples of EGFP^+^ cells (group 1) and three samples of EGFP^-^ cells (group 2). Each sample contained 300 sorted cells.

### RNA extraction

Six samples (three EGFP^+^ and three EGFP^-^) were used for total RNA extraction using the RNeasy Mini Kit (Qiagen, Redwood City, CA) according to manufacturer’s protocol. Additionally, total RNA from six samples of the whole head of WT larvae (group 3) was purified using the same kit. Each sample contained 60 heads. The quality and quantity of each RNA sample were assessed by Agilent's 2100 Bioanalyzer **6000** Pico Kit (Agilent Technologies, Santa Clara, CA).

### cDNA synthesis and amplification

RNA of group 1 and 2 (Figure 1I) was amplified using the Ovation® RNA-Seq System V2 (NuGEN, San Carlos, CA). Before amplification, all samples were lyophilized using a SpeedVac instrument and then suspended in 5 μl of nuclease-free water. The cDNAs were fragmented using a Bioruptor instrument with three 10 s (‘on’) cycles of sonication interrupted by 90 s pause periods (‘off’). The cDNAs of group 3 (Figure 1I) was synthesized according to standard procedures. The cDNAs were quantified using the Nanodrop and Bioanalyzer DNA 1000 Chip. The libraries were loaded on a High Sensitivity ChIP and quantified on a Qubit instrument.

### Illumina sequencing and bioinformatic analysis

Illumina TruSeq protocol was used to prepare libraries from RNA samples. Twelve libraries (group 1,2,3, Figure 1I) were run on 2 lanes of an Illumina HiSeq2000 machine using the multiplexing strategy of the TruSeq protocol (*Institute of Applied Genomics, Udine, Italy*). An average of 24 million reads were obtained from EGFP^+^ RNA, 22 million reads from EGFP^-^ RNA, and 175 million reads from the whole head RNA (http://www.ncbi.nlm.nih.gov/sra, PRJNA283169). Because of the difference in the amounts of RNA and the amplification process, the reads were 2×50 base pairs for the EGFP^+^ and EGFP^-^ groups, and 2×100 for whole larva head groups. The RNA-seq data from the replicates were unified, obtaining three groups for further analysis: EGFP^+^, EGFP^-^, and whole head groups. Since the amount of cells and RNA was very low, this strategy increased the read cover for each gene and resolved potential amplification bias. Cufflinks and Cuffdiffs (http://cufflinks.cbcb.umd.edu/) (Trapnell et al., 2010; Trapnell et al., 2012) were used to calculate gene-expression levels and identify differentially expressed transcripts (statistical analysis is described below). The reads were mapped to the zebrafish genome (Zv9), and raw read counts were normalized to TPM. Initially, a gene was considered to be preferentially expressed in the Hcrt cells if its normalized expression level was at least 100 TPM in EGFP^+^ cells and sevenfold higher than the higher of the normalized expression levels estimated in the two controls. For reference, the *hcrt*, which was expressed at a level above 300 TPM in the EGFP^+^ sample and below 10 TPM in the control samples, was used for aligning the reads against the zebrafish genome, allowing only uniquely aligned reads. In order to enlarge the list of enriched transcripts in Hcrt neurons, relaxed parameters were set and the new requirements were 10 TPM and 3.6 times higher abundance in EGFP^+^ cells compared with the control groups.

### Gene ontology analysis and prediction of transcription factors

Following analysis of the RNA-seq data, transcripts that were enriched in Hcrt neurons were either assigned to an annotated zebrafish gene or regarded as novel transcripts. To further characterize the annotated genes, they were assigned a human ortholog using the 'Non-Zebrafish RefSeq Genes' or the 'Human Proteins Mapped by Chained tBLASTn' tracks on the UCSC genome browser (Zv9/danRer7 assembly; http://genome.ucsc.edu/). To find over-represented molecular functions, human orthologs were used as input to DAVID annotation. We focused on over-represented TFs. The DAVID default human-gene background was used. All the significantly enriched (Benjamini-Hochberg adjusted *p*<0.05) TFs are presented in Figure 5—source data 1. The conserved location of transcription factor binding sites was identified in mammalian alignments. A binding site was considered to be conserved across the alignment if its score met the threshold score for its binding matrix. The score and threshold were calculated using the Transfac Matrix Database (v7.0) created by Biobase (Waltham, MA).

### Real-time quantitative PCR

The expression levels of *kcnh4a* mRNA were determined using quantitative real-time PCR. Total mRNA was extracted from *kcnh4a^-/-^* (n=9 batches of 8 larvae) and *kcnh4a^+/+^* (n=5 batches of 8 larvae) 6 dpf larvae, using the RNeasy Protect Mini Kit (Qiagen, Redwood City, CA) and according to the manufacturer's instructions. A similar amount of mRNA (1µg) was reverse-transcribed using Oligo (dT) oligos and SuperScript III reverse transcriptase (Invitrogen, Carlsbad, CA) according to the manufacturer's protocol. Transcript levels were determined by Applied Biosystems 7900HT Fast Real-Time PCR System using the Quanta SYBR FAST qPCR Kit (Quanta Biosciences, Gaithersburg, MD). *Ef1a* was used as reference gene (Tang et al., 2007) and ΔΔC_T_ analysis was performed (Schmittgen and Livak, 2008).

### PCR amplification and cloning of candidate genes

To prepare probes for whole-mount ISH experiments, the full coding sequences of the following genes were amplified: *hcrt* (NM_001077392.2), *star* (NM_131663.1), *dennd1b* (XM_009296374.1), *kcnh4a* (KR733682), *fam46a* (XM_005157860.2), *npvf* (NM_001082949.1), *npffr* (NM_001171697.1), *zgc:171844* (NM_001127478.1), *hmx3* (NM_131634.1), *lhx9* (NM_001017710.1), *si:dkey-58b18.8* (ENSDARG00000095761), *elovl7b* (NM_199778.1), *gad67* (NM_194419.1), *vglut2b* (NM_001009982.1), *hsf1* (NM_131600.1) and *cuff23873*. All PCR products were cloned into a pCRII-TOPO vector (Invitrogen, Carlsbad, CA) and served as a template to transcribe digoxigenin-labeled antisense mRNA probes.

### In-situ hybridization

Larvae and adult brains were fixed in 4% paraformaldehyde over 48 hr at 4°C. All samples were then dehydrated in 100% methanol and stored at -20°C. Before further treatment, brains and larvae were rehydrated in decreasing methanol concentrations. Adult brains were embedded in 2.5% agarose and sectioned with the Vibratome Series 1000 Sectioning System (Campden Instruments, Lafayette, IN). Transverse sections were then processed and stained as free-floating slices. ISH was performed following standard protocols. Digoxigenin- and fluorescein-labeled antisense riboprobes were transcribed in vitro using RNA Labeling Kit SP6/T7 (Roche Diagnostics Corporation, Indianapolis, IN). Single probe ISH was revealed with colorimetric BM purple (Roche Diagnostics Corporation, Indianapolis, IN). Double probe fluorescent ISH was performed as described previously (Levitas-Djerbi et al., 2015).

### **F**luorescent ISH and immunofluorescence staining

ISH was performed as described above in *hcrt:EGFP* 2 dpf larvae and adults. The samples were revealed using Fast Red (Roche Diagnostics Corporation, Indianapolis, IN). All the procedures were based on standard protocols (Levitas-Djerbi et al., 2015). After blocking, larvae and adult brain slices were incubated in primary rabbit anti-EGFP (Santa Cruz Biotechnology, Santa Cruz, CA), diluted 1:250. Anti-EGFP antibodies were detected with a secondary goat anti-rabbit Alexa Fluor 488 IgG (H L) antibody (2 mg/mL, A-11034, Invitrogen, Carlsbad, CA). All experiments were repeated in 3-–5 larvae and adult sections.

### Imaging

An epifluorescence stereomicroscope (Leica M165FC) was used to visualize live larvae expressing EGFP and fluorescent-fixed larvae and adult brain sections. Pictures were taken using the Leica Application Suite imaging software, version 3.7 (Leica, Wetzlar, Germany). In confocal imaging of fixed embryos, the samples were mounted on slides. In live imaging of *hcrt:EGFP* larvae, the larvae were mounted in low-melting–point 1% agarose. Confocal imaging was performed using a Zeiss LSM710 upright confocal microscope (Zeiss, Oberkochen, Germany). All images were processed using ImageJ (National Institutes of Health, Bethesda, MD).

### Establishment of a *kcnh4a* mutant (*kcnh4a*^-/-^) line

The CRISPR system (Hwang et al., 2013) was used to establish the *kcnh4a*^-/-^ line. The Cas9 (Addgene plasmid no. 42251) and sgRNA (Addgene plasmid no. DR274) zebrafish expression plasmids were obtained from Addgene (Cambridge, MA). In order to prepare the sgRNA, two *kcnh4a*-specific oligos were designed to match the target site (ACAACGTCTGCTTCTCCACCC) in exon 5. These oligos were denatured at 95°C for 5 min, then gradually cooled down to room temperature and kept at 4o°C. Before cloning, annealing of the oligos was confirmed in 2% agarose gel. The annealed oligos were cloned into the DR274 plasmid between the *BsaI* restriction sites and transformed into bacteria, which was selected by standard sequencing. In order to synthesize the specific sgRNA, the DR274 plasmid containing the annealed oligos was linearized with the restriction enzyme *DraI*, and cleaned using the standard phenol-chloroform procedure, followed by purification by the PureLink PCR Purification Kit (#K31000, Life Technologies, Carlsbad, CA). The sgRNA was synthesized using the T7 High Yield RNA Synthesis Kit (New England Biolabs, Hitchin, UK). In order to prepare *Cas9* mRNA, the zebrafish Cas9 vector was linearized by *AgeI*, and mRNA was synthesized using the mMESSAGE mMACHINE T7 Kit (Life Technologies, Carlsbad, CA).

One-cell–stage WT zebrafish embryos were microinjected with mixed *Cas9* mRNA (300 ng/µl) and transcribed sgRNA (12.5 ng/µl). To test the efficiency of the CRISPR system, ten 1-dpf embryos were screened for *kcnh4a*-specific mutation (as described below). We found that 60% of the embryos carried the mutation. The founder (F0) mosaic embryos were raised to adulthood and outcrossed with WT fish in order to identify F1 mutant fish. Single F1 heterozygous fish, which carry a 14 bp deletion mutation in the *kcnh4a* target site (Figure 6D), was selected and outcrossed with WT fish. To decrease the risk for off-target mutations, heterozygous F2 and then F3 fish were outcrossed with WT fish. In all experiments, heterozygous F4 fish were intercrossed, and the assays were performed on their progeny.

### Genotyping

Genotyping of the *kcnh4a*^-/-^ zebrafish was conducted by extracting genomic DNA from embryos and larvae or by the tail clipping of adult fish using the KAPA Express Extract Kit (Kapa Biosystems Inc., Boston, MA) according to the manufacturer's instructions. Genomic DNA was then amplified by PCR using the following primers: forward- 5^'^TTCATGTTTTCCACAGAATGTGTTTTCACA3^'^ and reverse- 5^'^ACCGAGGATGAAGAGCATCTCCACAG3^'^. The PCR product was then run on 2% agarose gel, and heterozygous, homozygous, and WT fragments could be identified by their size (Figure 6D). To confirm the gel pattern, selected PCR products were sequenced.

### Behavioral assays

The *kcnh4a*^+/−^ adult zebrafish were intercrossed and their progeny were kept under LD cycle. At 5 dpf, the larvae were individually placed in 48-well plates. At 6 dpf, larva-containing plates were placed in the Noldus DanioVision tracking system (Noldus Information Technology, Wageningen, Netherlands) and acclimated for one hour prior to behavioral recording. Recording was performed using the EthoVision XT 9 software (Noldus Information Technology, Wageningen, Netherlands), as previously described (Elbaz et al., 2012). Light intensity in the tracking system was 70 LUX for all experiments. To monitor rhythmic behavior during a daily cycle, larvae were maintained under the LD cycle, which was similar to the LD cycle prior to the experiment. Data analyses of total locomotor activity, sleep time, sleep\wake transitions, and sleep-bout length were performed according to the parameters previously described (Elbaz et al., 2012). Following each behavioral experiment, all larvae were subject to genotyping (as described above). SD was performed by randomized manual tapping on a petri dish that contained 6 dpf larvae. Following the SD, sleep time was monitored in sleep-deprived and control larvae (n = 13 for each treatment) using behavioral systems.

### Statistical analysis

In the RNA-seq data, statistically significant differences between the number of reads aligned to each gene (the expression profile) in the different tested conditions, without unifying the replicates, were identified as previously described (Alon et al., 2011; Ben-Moshe et al., 2014). Briefly, the expression profiles were normalized using a variation of the trimmed mean of M-values normalization method (Robinson and Oshlack, 2010; Alon et al., 2011). Subsequently, we searched for expression differences between the EGFP^+^ and the control samples that cannot be explained by Poisson noise with *p* <0.01 and Bonferroni correction for multiple testing (Alon et al., 2011). Notably, the analysis takes into account technical biases that can cause the variance to be larger than that of naive Poisson statistics (Alon et al., 2011). Only genes with average expression >15 (raw reads) in the EGFP^+^ samples were analyzed, and only genes with fold change higher than 3.6 are shown in Figure 2—source data 1.

In the behavioral experiments, statistical analysis was performed using SAS v9.3 software (SAS Institute, Cary, NC). Locomotor activity, sleep time, sleep-bout length, and sleep\wake transitions were analyzed with repeated measures of ANOVA (SAS PROC MIXED), where each was modeled as a function of genotype (*kcnh4a^-/-^, kcnh4a^+/-^, kcnh4a^+/+^*), time (24 hr), and the genotype by time interaction term. LS means (model estimated means) differences between the genotype groups per time point were estimated from the model interaction terms and are presented with respective levels of significance and 95% confidence intervals. These were used to compare between genotypes per time point in locomotor activity and sleep experiments.

## Additional files

### Major datasets

The following datasets were generated:

Yelin-Bekerman L, Appelbaum L, 2015,Bioproject: Transcriptional profiling of hypocretin neurons identifies the sleep regulator Kcnh4a, http://www.ncbi.nlm.nih.gov/sra/?term=PRJNA283169, Publicly available at the NCBI Short Read Archive (Accession no: PRJNA283169)
