# Supplementary material for: Hypocretin neuron-specific transcriptome profiling identifies the sleep modulator Kcnh4a
Source: eLife. 2015 Oct 1;4:e08638. doi: 10.7554/eLife.08638 (PMC4718730; doi:10.7554/eLife.08638)
Supplement: Figure 2—source data 1. — All the genes detected have p <0.01 with Bonferroni correction for multiple testing (Materials and methods). DOI: http://dx.doi.org/10.7554/eLife.08638.005 [file elife-08638-fig2-data1.docx]

| Transcript  Accession ID | Transcript  Name | Fold Change | Human  Orthologous gene |
| --- | --- | --- | --- |
| ENSDART00000052627 | *npvf* | 362.4 | *npvf* |
| ENSDART00000104549 | *hcrt* | 54.2 | *hcrt* |
| cuff.23873 | *cuff.23873* | 28.4 |  |
| cuff.70256 | *cuff.70256* | 27.9 |  |
| cuff.46280 | *cuff.46280* | 21.3 |  |
| ENSDART00000090633 | *kcnh4a* | 20.2 | *kcnh4* |
| cuff.64723 | *cuff.64723* | 15.1 |  |
| ENSDART00000010028 | *ptgs2b* | 14.5 | *ptgs2* |
| ENSDART00000014385 | *elovl7b* | 13.5 | *elovl7* |
| ENSDART00000111870 | *zacn* | 11.3 |  |
| cuff.34876 | *cuff.34876* | 10.8 |  |
| cuff.57637 | *cuff.57637* | 10.5 |  |
| ENSDART00000016225 | *star* | 10.5 | *star* |
| ENSDART00000144905 | *vgll2a* | 10.3 | *vgll2* |
| ENSDART00000030938 | *adra1a* | 10.2 | *adra1a* |
| ENSDART00000079150 | *grpr* | 9.5 | *grpr* |
| ENSDART00000127907 | *wu:fc34e06* | 9.3 |  |
| cuff.42205 | *cuff.42205* | 9.3 |  |
| cuff.42204 | *cuff.42204* | 8.9 |  |
| ENSDART00000144655 | *si:dkey-58b18.8* | 8.4 |  |
| cuff.42203 | *cuff.42203* | 8.2 |  |
| ENSDART00000129866 | *pcsk2* | 8.2 | *pcsk2* |
| ENSDART00000105614 | *dennd1b* | 8.0 | *dennd1b* |
| ENSDART00000109144 | *ptprnb* | 7.9 | *ptprn* |
| cuff.77494 | *cuff.77494* | 7.9 |  |
| cuff.64722 | *cuff.64722* | 7.8 |  |
| cuff.69728 | *cuff.69728* | 7.6 |  |
| ENSDART00000135731 | *npffr1l2* | 7.5 | *npffr1* |
| cuff.77484 | *cuff.77484* | 7.3 |  |
| cuff.77481 | *cuff.77481* | 7.3 |  |
| ENSDART00000054071 | *fam46a* | 7.2 | *fam46a* |
| ENSDART00000141267 | *si:ch211-199o1.2* | 7.1 | *hspa1l* |
| ENSDART00000148896 | *rfx4* | 7.0 | *rfx4* |
| cuff.16569 | *cuff.16569* | 7.0 |  |
| cuff.8734 | *cuff.8734* | 6.9 |  |
| ENSDART00000083582 | *soat2* | 6.7 | *soat2* |
| ENSDART00000087586 | *c2cd4a* | 6.7 | *c2cd4a* |
| cuff.70259 | *cuff.70259* | 6.6 |  |
| ENSDART00000141006 | *crb1* | 6.5 | *crb1* |
| ENSDART00000149967 | *scg2b* | 6.3 | *scg2* |
| cuff.77491 | *cuff.77491* | 6.1 |  |
| ENSDART00000114023 | *hspb9* | 6.1 |  |
| cuff.11863 | *cuff.11863* | 6.0 |  |
| ENSDART00000111193 | *cntnap5* | 6.0 |  |
| ENSDART00000136049 | *syt10* | 5.9 | *syt10* |
| cuff.70259 | *cuff.70259* | 5.9 |  |
| cuff.85986 | *CU466240.1* | 5.6 |  |
| ENSDART00000041319 | *slc4a2a* | 5.6 | *slc4a1* |
| cuff.23006 | *cuff.23006* | 5.5 |  |
| ENSDART00000079556 | *lhx9* | 5.4 | *lhx9* |
| ENSDART00000125953 | *ttnb* | 5.4 | *ttn* |
| ENSDART00000143847 | *ptprna* | 5.3 | *ptprn* |
| ENSDART00000113112 | *wscd1* | 5.3 | *wscd1* |
| ENSDART00000103506 | *hpcal4* | 5.3 | *hpcal4* |
| ENSDART00000140291 | *si:dkey-203c4.2* | 5.2 |  |
| cuff.9352 | *cuff.9352* | 5.2 |  |
| cuff.32637 | *cuff.32637* | 5.2 |  |
| cuff.18118 | *cuff.18118* | 5.1 |  |
| ENSDART00000112043 | *cacng4* | 5.0 | *cacng4* |
| ENSDART00000119540 | *5S_rRNA* | 5.0 |  |
| cuff.60512 | *cuff.60512* | 5.0 |  |
| cuff.3221 | *cuff.3221* | 4.9 |  |
| cuff.7864 | *cuff.7864* | 4.9 |  |
| ENSDART00000055659 | *cyb561* | 4.8 | *cyb561* |
| cuff.78817 | *cuff.78817* | 4.8 |  |
| ENSDART00000046891 | *cetp* | 4.7 | *cetp* |
| cuff.3779 | *cuff.3779* | 4.7 |  |
| cuff.8414 | *cuff.8414* | 4.7 |  |
| cuff.30543 | *cuff.30543* | 4.6 |  |
| ENSDART00000102681 | *pnp5a* | 4.6 | *pnp* |
| cuff.11620 | *cuff.11620* | 4.6 |  |
| cuff.73756 | *cuff.73756* | 4.6 |  |
| cuff.19765 | *cuff.19765* | 4.6 |  |
| ENSDART00000133186 | *ntng1a* | 4.5 | *ntng1* |
| ENSDART00000003653 | *scg2a* | 4.5 |  |
| cuff.10192 | *cuff.10192* | 4.5 |  |
| cuff.6591 | *cuff.6591* | 4.5 |  |
| ENSDART00000011573 | *mmp13a* | 4.5 | *mmp13* |
| cuff.35871 | *cuff.35871* | 4.5 |  |
| cuff.58718 | *cuff.58718* | 4.5 |  |
| cuff.50000 | *cuff.50000* | 4.5 |  |
| cuff.61171 | *cuff.61171* | 4.4 |  |
| cuff.35077 | *cuff.35077* | 4.4 |  |
| cuff.24429 | *cuff.24429* | 4.4 |  |
| cuff.54951 | *cuff.54951* | 4.4 |  |
| ENSDART00000124859 | *pde2a* | 4.4 | *pde2a* |
| cuff.25645 | *cuff.25645* | 4.3 |  |
| cuff.30643 | *cuff.30643* | 4.3 |  |
| cuff.50831 | *cuff.50831* | 4.3 |  |
| cuff.77495 | *cuff.77495* | 4.3 |  |
| cuff.83598 | *cuff.83598* | 4.3 |  |
| ENSDART00000088907 | *npas4* | 4.3 |  |
| cuff.65431 | *cuff.65431* | 4.3 |  |
| cuff.8136 | *cuff.8136* | 4.3 |  |
| cuff.54414 | *cuff.54414* | 4.3 |  |
| cuff.43751 | *cuff.43751* | 4.3 |  |
| cuff.82781 | *cuff.82781* | 4.2 |  |
| cuff.13810 | *cuff.13810* | 4.2 |  |
| cuff.86048 | *cuff.86048* | 4.2 |  |
| ENSDART00000145508 | *igfbp1a* | 4.2 | *igfbp4* |
| cuff.68809 | *cuff.68809* | 4.2 |  |
| cuff.81658 | *cuff.81658* | 4.2 |  |
| cuff.11284 | *cuff.11284* | 4.2 |  |
| cuff.8308 | *cuff.8308* | 4.2 |  |
| ENSDART00000128945 | *cabzo1048956.1* | 4.2 |  |
| cuff.53719 | *cuff.53719* | 4.2 |  |
| ENSDART00000011471 | *dhrs13a.2* | 4.2 |  |
| cuff.12935 | *cuff.12935* | 4.1 |  |
| cuff.52026 | *cuff.52026* | 4.1 |  |
| ENSDART00000114982 | *f2rl1.2* | 4.1 | *f2rl1* |
| cuff.8383 | *cuff.8383* | 4.1 |  |
| cuff.74840 | *cuff.74840* | 4.1 |  |
| cuff.27060 | *cuff.27060* | 4.1 |  |
| cuff.50876 | *cuff.50876* | 4.1 |  |
| cuff.48281 | *cuff.48281* | 4.1 |  |
| cuff.63288 | *cuff.63288* | 4.1 |  |
| ENSDART00000113247 | *tsen54* | 4.1 | *tsen54* |
| cuff.84476 | *cuff.84476* | 4.0 |  |
| ENSDART00000045905 | *cabz01064228.2* | 4.0 | *trpc7* |
| cuff.25067 | *cuff.25067* | 4.0 |  |
| cuff.45488 | *cuff.45488* | 4.0 |  |
| cuff.19767 | *cuff.19767* | 4.0 |  |
| ENSDART00000063175 | *cabz01069335.1* | 4.0 | *mcoln3* |
| ENSDART00000104612 | *hmx3* | 4.0 | *hmx3* |
| cuff.49005 | *cuff.49005* | 4.0 |  |
| ENSDART00000056453 | *igfbp1b* | 4.0 |  |
| ENSDART00000097679 | *zgc:171844* | 4.0 | *c16orf45* |
| cuff.21343 | *cuff.21343* | 4.0 |  |
| cuff.45503 | *cuff.45503* | 4.0 |  |
| cuff.49702 | *cuff.49702* | 4.0 |  |
| cuff.64685 | *cuff.64685* | 4.0 |  |
| cuff.77482 | *cuff.77482* | 4.0 |  |
| ENSDART00000025428 | *epha2* | 4.0 | *epha2* |
| ENSDART00000109597 | *C2cd4c* | 4.0 |  |
| cuff.40728 | *cuff.40728* | 4.0 |  |
| cuff.1806 | *cuff.1806* | 4.0 |  |
| ENSDART00000028265 | *nr5a1b* | 4.0 | *nr5a1* |
| cuff.19760 | *cuff.19760* | 3.9 |  |
| cuff.72401 | *cuff.72401* | 3.9 |  |
| cuff.71766 | *cuff.71766* | 3.9 |  |
| cuff.68816 | *cuff.68816* | 3.9 |  |
| cuff.77202 | *cuff.77202* | 3.9 |  |
| cuff.27392 | *cuff.27392* | 3.9 |  |
| cuff.27544 | *cuff.27544* | 3.9 |  |
| cuff.3962 | *cuff.3962* | 3.9 |  |
| ENSDART00000099810 | *nos1* | 3.9 | *nos1* |
| cuff.1516 | *cuff.1516* | 3.9 |  |
| cuff.50313 | *cuff.50313* | 3.9 |  |
| cuff.51762 | *cuff.51762* | 3.9 |  |
| cuff.48931 | *cuff.48931* | 3.9 |  |
| ENSDART00000010449 | *ugp2a* | 3.9 | *ugp2* |
| ENSDART00000139527 | *si:ch211-59d15.4* | 3.9 |  |
| cuff.16817 | *cuff.16817* | 3.9 |  |
| cuff.5575 | *cuff.5575* | 3.9 |  |
| cuff.13165 | *cuff.13165* | 3.9 |  |
| cuff.43612 | *cuff.43612* | 3.9 |  |
| cuff.1247 | *cuff.1247* | 3.8 |  |
| cuff.23897 | *cuff.23897* | 3.8 |  |
| cuff.26108 | *cuff.26108* | 3.8 |  |
| ENSDART00000148685 | *xirp1* | 3.8 | *cmya1* |
| cuff.23008 | *cuff.23008* | 3.8 |  |
| cuff.25991 | *cuff.25991* | 3.8 |  |
| cuff.23624 | *cuff.23624* | 3.8 |  |
| cuff.71533 | *cuff.71533* | 3.8 |  |
| ENSDART00000128834 | *muc22* | 3.8 |  |
| cuff.25733 | *cuff.25733* | 3.8 |  |
| cuff.30924 | *cuff.30924* | 3.8 |  |
| cuff.31982 | *cuff.31982* | 3.8 |  |
| cuff.8149 | *cuff.8149* | 3.8 |  |
| cuff.22844 | *cuff.22844* | 3.8 |  |
| cuff.21953 | *cuff.21953* | 3.8 |  |
| cuff.30026 | *cuff.30026* | 3.8 |  |
| cuff.36357 | *cuff.36357* | 3.8 |  |
| cuff.45076 | *cuff.45076* | 3.8 |  |
| cuff.4047 | *cuff.4047* | 3.8 |  |
| ENSDART00000024330 | *creb3l1* | 3.8 | *creb3l1* |
| cuff.6715 | *cuff.6716* | 3.8 |  |
| ENSDART00000047841 | *sgsm1* | 3.8 | *sgsm1* |
| cuff.40136 | *cuff.40136* | 3.8 |  |
| cuff.64182 | *cuff.64182* | 3.7 |  |
| cuff.74494 | *cuff.74494* | 3.7 |  |
| cuff.17329 | *cuff.17329* | 3.7 |  |
| cuff.59352 | *cuff.59352* | 3.7 |  |
| cuff.32410 | *cuff.32410* | 3.7 |  |
| cuff.10958 | *cuff.10958* | 3.7 |  |
| cuff.42713 | *cuff.42713* | 3.7 |  |
| cuff.50246 | *cuff.50246* | 3.7 |  |
| cuff.68184 | *cuff.68184* | 3.7 |  |
| cuff.77962 | *cuff.77962* | 3.7 |  |
| cuff.8591 | *cuff.8591* | 3.7 |  |
| cuff.74574 | *cuff.74574* | 3.7 |  |
| ENSDART00000101418 | *chrm5b* | 3.7 |  |
| cuff.77166 | *cuff.77166* | 3.7 |  |
| cuff.84348 | *cuff.84348* | 3.7 |  |
| cuff.16089 | *cuff.16089* | 3.7 |  |
| cuff.10953 | *cuff.10953* | 3.7 |  |
| cuff.4378 | *cuff.4378* | 3.7 |  |
| cuff.71568 | *cuff.71568* | 3.7 |  |
| ENSDART00000124669 | *npas4b* | 3.7 |  |
| cuff.45104 | *cuff.45104* | 3.7 |  |
| cuff.45934 | *cuff.45934* | 3.7 |  |
| cuff.20157 | *cuff.20157* | 3.7 |  |
| cuff.62635 | *cuff.62635* | 3.6 |  |
| ENSDART00000133136 | *si:dkey-238o14.2* | 3.6 |  |
| cuff.77483 | *cuff.77483* | 3.6 |  |
| cuff.45040 | *cuff.45040* | 3.6 |  |
| cuff.9215 | *cuff.9215* | 3.6 |  |
| cuff.3229 | *cuff.3229* | 3.6 |  |
| cuff.66548 | *cuff.66548* | 3.6 |  |
| cuff.19344 | *cuff.19344* | 3.6 |  |
| cuff.64308 | *cuff.64308* | 3.6 |  |
| cuff.85999 | *cuff.85999* | 3.6 |  |
