# Supplementary material for: Hypocretin neuron-specific transcriptome profiling identifies the sleep modulator Kcnh4a
Source: eLife. 2015 Oct 1;4:e08638. doi: 10.7554/eLife.08638 (PMC4718730; doi:10.7554/eLife.08638)
Supplement: Figure 5—source data 1. — DOI: http://dx.doi.org/10.7554/eLife.08638.009 [file elife-08638-fig5-data1.docx]

| Transcription Factor  Table S2: Predicted Hcrt-neuron enriched transcription factors and their target genes | Number of predicted regulated transcripts | *p*-value | Predicted regulated transcripts |
| --- | --- | --- | --- |
| *pax4* | 44 | 9.81E-05 | *hcrt, ptgs2, fam46a, ttn, pnp, pcsk2, hspa1l, mcoln3, crb1, grpr, creb3l1,*  *elovl7, cetp, krt4, npffr1, slc4a1, lhx9, c16orf45, scg2, soat2, tsen54, nos1,*  *rfx4, syt10, hpcal4, trpc7, ntng1, cacng4, myh4, ptprn, cyb561, epha2,*  *dennd1b, npvf, wscd1, pde2a, adra1a, vgll2, c2cd4a, hmx3, kcnh4a, ugp2,*  *igfbp4, nr5a1* |
| *hsf1* | 25 | 1.09E-04 | *hcrt, ptgs2, ttn, hspa1l, grpr, elovl7, slc4a1, lhx9, c16orf45, soat2, tsen54,*  *nos1, rfx4, syt10, trpc7, ntng1, myh4, cacng4, dennd1b, sgsm1, pde2a,*  *wscd1, adra1a, kcnh4a, hmx3* |
| *hnf1* | 35 | 3.57E-04 | *ptgs2, f2rl1, ttn, fam46a, pcsk2, mcoln3, crb1, grpr, creb3l1, elovl7, lhx9,*  *c16orf45, scg2, soat2, nos1, rfx4, syt10, hpcal4, trpc7, ntng1, cacng4,*  *myh4, mmp13, cyb561, epha2, dennd1b, npvf, sgsm1, wscd1, pde2a,*  *adra1a, vgll2, hmx3, ugp2, igfbp4* |
| *ap2* | 13 | 4.57E-04 | *hcrt, rfx4, ttn, pcsk2, pde2a, wscd1, mcoln3, creb3l1, slc4a1, lhx9, kcnh4a,*  *hmx3, nr5a1* |
| *pou6f1* | 29 | 6.29E-04 | *star, ptgs2, ttn, fam46a, pnp, pcsk2, mcoln3, crb1, grpr, creb3l1, elovl7,*  *lhx9, c16orf45, syt10, trpc7, ntng1, cacng4, mmp13, cyb561, dennd1b,*  *npvf, sgsm1, wscd1, vgll2, adra1a, hmx3, ugp2, igfbp4, nr5a1* |
| *ap1* | 36 | 8.36E-04 | *ttn, pnp, hspa1l, pcsk2, mcoln3, crb1, grpr, creb3l1, elovl7, npffr1, slc4a1,*  *krt4, lhx9, c16orf45, scg2, soat2, tsen54, nos1, rfx4, hpcal4, trpc7, ntng1,*  *cacng4, myh4, ptprn, mmp13, cyb561, epha2, dennd1b, npvf, sgsm1,*  *wscd1, pde2a, adra1a, igfbp4, nr5a1* |
| *chx10* | 28 | 0.001288 | *hcrt, ttn, fam46a, pcsk2, crb1, grpr, creb3l1, lhx9, scg2, nos1, rfx4, syt10,*  *trpc7, ntng1, cacng4, myh4, cyb561, epha2, dennd1b, npvf, pde2a, wscd1,*  *vgll2, adra1a, hmx3, ugp2, igfbp4, kcnh4a* |
| *ppara* | 33 | 0.001779 | *hcrt, ptgs2, ttn, fam46a, pnp, hspa1l, pcsk2, crb1, grpr, creb3l1, npffr1,*  *slc4a1, krt4, lhx9, soat2, tsen54, nos1, rfx4, syt10, trpc7, ntng1, cacng4,*  *myh4, ptprn, cyb561, epha2, sgsm1, pde2a, adra1a, hmx3, igfbp4, kcnh4a,*  *nr5a1* |
| *gcnf* | 34 | 0.002021 | *hcrt, f2rl1, ttn, fam46a, pnp, hspa1l, pcsk2, mcoln3, crb1, grpr, creb3l1,*  *slc4a1, npffr1, krt4, scg2, soat2, nos1, rfx4, trpc7, ntng1, cacng4, myh4,*  *ptprn, epha2, dennd1b, npvf, sgsm1, wscd1, pde2a, adra1a, c2cd4a,*  *hmx3, igfbp4, kcnh4a* |
| *mycmax* | 36 | 0.00237 | *hcrt, star, fam46a, ttn, pnp, pcsk2, hspa1l, crb1, cetp, npffr1, slc4a1, lhx9,*  *c16orf45, scg2, soat2, nos1, rfx4, syt10, hpcal4, trpc7, ntng1, cacng4,*  *myh4, ptprn, cyb561, epha2, dennd1b, npvf, sgsm1, wscd1, pde2a, c2cd4a,*  *hmx3, igfbp4, kcnh4a, nr5a1* |
| *lhx3* | 25 | 0.002713 | *f2rl1, ttn, fam46a, pcsk2, crb1, grpr, elovl7, lhx9, c16orf45, scg2, nos1,*  *rfx4, syt10, trpc7, ntng1, myh4, cacng4, epha2, dennd1b, npvf, adra1a,*  *c2cd4a, igfbp4, ugp2, nr5a1* |
| *taxcreb* | 32 | 0.003951 | *hcrt, f2rl1, ttn, pnp, hspa1l, pcsk2, grpr, creb3l1, npffr1, slc4a1, krt4,*  *tsen54, nos1, rfx4, trpc7, ntng1, cacng4, myh4, ptprn, cyb561, epha2,*  *sgsm1, wscd1, pde2a, adra1a, vgll2, c2cd4a, hmx3, ugp2, igfbp4,*  *kcnh4a, nr5a1* |
| *foxo4* | 32 | 0.004027 | *ptgs2, ttn, fam46a, hspa1l, pcsk2, mcoln3, crb1, grpr, slc4a1, lhx9,*  *c16orf45, soat2, nos1, rfx4, syt10, hpcal4, trpc7, ntng1, cacng4, myh4,*  *ptprn, mmp13, cyb561, dennd1b, sgsm1, wscd1, pde2a, adra1a, c2cd4a,*  *ugp2, igfbp4, nr5a1* |
| *s8* | 28 | 0.005508 | *hcrt, ttn, fam46a, pnp, pcsk2, crb1, grpr, elovl7, lhx9, scg2, nos1, rfx4,*  *syt10, trpc7, ntng1, myh4, cacng4, mmp13, epha2, dennd1b, pde2a,*  *wscd1, vgll2, adra1a, hmx3, ugp2, kcnh4a, nr5a1* |
| *tcf11* | 30 | 0.006451 | *hcrt, star, ttn, pnp, pcsk2, crb1, grpr, creb3l1, npffr1, slc4a1, c16orf45,*  *scg2, soat2, nos1, rfx4, syt10, trpc7, ntng1, cacng4, myh4, ptprn, cyb561,*  *epha2, dennd1b, sgsm1, pde2a, adra1a, hmx3, kcnh4a, nr5a1* |
| *hsf2* | 26 | 0.007329 | *hcrt, ttn, fam46a, hspa1l, pcsk2, crb1, mcoln3, grpr, lhx9, c16orf45, scg2,*  *trpc7, ntng1, myh4, cacng4, epha2, dennd1b, npvf, wscd1, vgll2, adra1a,*  *c2cd4a, hmx3, ugp2, kcnh4a, nr5a1* |
| *nf1* | 24 | 0.007423 | *hcrt, tsen54, nos1, rfx4, star, hpcal4, myh4, ttn, fam46a, pnp, cyb561,*  *epha2, hspa1l, pcsk2, crb1, pde2a, creb3l1, adra1a, slc4a1, krt4, lhx9,*  *igfbp4, kcnh4a, nr5a1* |
| *nfkappaab50* | 17 | 0.007487 | *nos1, rfx4, ntng1, cacng4, ptprn, fam46a, ttn, mmp13, epha2, dennd1b,*  *pcsk2, wscd1, creb3l1, lhx9, hmx3, igfbp4, nr5a1* |
| *htf* | 31 | 0.007561 | *hcrt, star, ttn, pcsk2, grpr, cetp, elovl7, slc4a1, c16orf45, scg2, soat2,*  *tsen54, nos1, rfx4, hpcal4, trpc7, ntng1, cacng4, myh4, ptprn, mmp13,*  *cyb561, epha2, dennd1b, sgsm1, pde2a, vgll2, adra1a, hmx3, kcnh4a,*  *nr5a1* |
| *e47* | 34 | 0.008063 | *hcrt, star, ttn, fam46a, hspa1l, pcsk2, crb1, grpr, creb3l1, elovl7, npffr1,*  *slc4a1, lhx9, c16orf45, soat2, tsen54, nos1, rfx4, hpcal4, trpc7, ntng1,*  *cacng4, myh4, ptprn, epha2, dennd1b, npvf, wscd1, pde2a, adra1a,*  *vgll2, hmx3, kcnh4a, nr5a1* |
| *tata* | 31 | 0.008432 | *hcrt, ttn, fam46a, pnp, pcsk2, mcoln3, crb1, grpr, creb3l1, lhx9, c16orf45,*  *nos1, rfx4, syt10, ntng1, cacng4, myh4, mmp13, cyb561, epha2, dennd1b,*  *npvf, sgsm1, wscd1, adra1a, c2cd4a, hmx3, ugp2, igfbp4, kcnh4a, nr5a1* |
| *nfe2* | 23 | 0.008497 | *soat2, nos1, rfx4, hpcal4, trpc7, ntng1, myh4, cacng4, ptprn, ttn, cyb561,*  *epha2, dennd1b, pcsk2, npvf, sgsm1, pde2a, grpr, creb3l1, c2cd4a,*  *igfbp4, kcnh4a, c16orf45* |
| *usf* | 33 | 0.008552 | *hcrt, ptgs2, ttn, fam46a, pnp, pcsk2, crb1, creb3l1, elovl7, npffr1, lhx9,*  *scg2, soat2, nos1, rfx4, hpcal4, trpc7, ntng1, myh4, cyb561, epha2,*  *dennd1b, npvf, sgsm1, wscd1, pde2a, vgll2, c2cd4a, hmx3, ugp2, igfbp4,*  *kcnh4a, nr5a1* |
| *rfx1* | 34 | 0.009556 | *hcrt, star, f2rl1, ttn, fam46a, pnp, hspa1l, mcoln3, crb1, grpr, elovl7,*  *slc4a1, krt4, scg2, tsen54, nos1, rfx4, syt10, hpcal4, trpc7, ntng1,*  *cacng4, cyb561, dennd1b, sgsm1, wscd1, pde2a, adra1a, c2cd4a,*  *hmx3, ugp2, igfbp4, kcnh4a, nr5a1* |
| *creb* | 26 | 0.010085 | *star, ttn, hspa1l, pcsk2, mcoln3, grpr, creb3l1, slc4a1, lhx9, scg2, nos1,*  *rfx4, hpcal4, ntng1, cacng4, ptprn, epha2, dennd1b, sgsm1, wscd1,*  *vgll2, adra1a, c2cd4a, hmx3, igfbp4, nr5a1* |
| *arnt* | 28 | 0.010417 | *hcrt, star, ttn, fam46a, pnp, grpr, cetp, elovl7, lhx9, soat2, tsen54, nos1,*  *rfx4, hpcal4, trpc7, ntng1, myh4, ptprn, cyb561, npvf, sgsm1, pde2a,*  *vgll2, adra1a, hmx3, igfbp4, kcnh4a, nr5a1* |
| *cdpcr3* | 31 | 0.010438 | *hcrt, ptgs2, ttn, fam46a, pnp, hspa1l, pcsk2, crb1, creb3l1, elovl7, npffr1,*  *lhx9, c16orf45, tsen54, nos1, rfx4, trpc7, ntng1, cacng4, myh4, ptprn,*  *cyb561, epha2, dennd1b, wscd1, pde2a, adra1a, hmx3, igfbp4, kcnh4a,*  *nr5a1* |
| *lun1* | 29 | 0.010875 | *star, ttn, pnp, pcsk2, creb3l1, slc4a1, npffr1, krt4, c16orf45, scg2,*  *tsen54, nos1, rfx4, hpcal4, ntng1, cacng4, myh4, ptprn, cyb561, epha2,*  *dennd1b, sgsm1, wscd1, pde2a, c2cd4a, hmx3, igfbp4, kcnh4a, nr5a1* |
| *irf2* | 25 | 0.011628 | *ttn, fam46a, pnp, pcsk2, crb1, creb3l1, elovl7, lhx9, soat2, tsen54, rfx4,*  *trpc7, ntng1, myh4, cacng4, cyb561, mmp13, dennd1b, npvf, wscd1,*  *vgll2, adra1a, ugp2, kcnh4a, hmx3* |
| *hox13* | 27 | 0.011773 | *hcrt, ttn, pnp, hspa1l, pcsk2, crb1, grpr, creb3l1, npffr1, slc4a1, krt4, lhx9,*  *c16orf45, tsen54, rfx4, trpc7, ntng1, myh4, ptprn, cyb561, epha2, dennd1b,*  *pde2a, wscd1, hmx3, kcnh4a, nr5a1* |
| *sox5* | 28 | 0.012542 | *ptgs2, f2rl1, ttn, pcsk2, mcoln3, crb1, grpr, creb3l1, elovl7, slc4a1, npffr1,*  *lhx9, tsen54, rfx4, syt10, ntng1, ptprn, cyb561, mmp13, dennd1b, npvf,*  *pde2a, adra1a, c2cd4a, hmx3, ugp2, igfbp4, nr5a1* |
| *gata1* | 41 | 0.01471 | *hcrt, star, f2rl1, fam46a, ttn, pnp, pcsk2, hspa1l, mcoln3, crb1, grpr,*  *creb3l1, elovl7, npffr1, slc4a1, lhx9, c16orf45, scg2, soat2, tsen54, nos1,*  *rfx4, syt10, trpc7, ntng1, myh4, ptprn, cyb561, epha2, dennd1b, npvf,*  *wscd1, pde2a, adra1a, vgll2, c2cd4a, hmx3, kcnh4a, ugp2, igfbp4, nr5a1* |
| *mzf1* | 28 | 0.014724 | *star, ttn, fam46a, pnp, hspa1l, pcsk2, crb1, creb3l1, npffr1, slc4a1, lhx9,*  *soat2, tsen54, nos1, rfx4, hpcal4, ntng1, cacng4, myh4, ptprn, cyb561,*  *epha2, dennd1b, sgsm1, pde2a, ugp2, igfbp4, nr5a1* |
| *pax2* | 32 | 0.017324 | *hcrt, ttn, pnp, hspa1l, pcsk2, mcoln3, creb3l1, elovl7, npffr1, slc4a1,*  *c16orf45, soat2, tsen54, nos1, rfx4, syt10, hpcal4, trpc7, ntng1, cacng4,*  *myh4, ptprn, epha2, dennd1b, npvf, sgsm1, wscd1, pde2a, vgll2, ugp2,*  *kcnh4a, nr5a1* |
| *tal1betae47* | 19 | 0.019655 | *hcrt, tsen54, ntng1, cacng4, ptprn, ttn, dennd1b, pcsk2, pde2a, crb1,*  *wscd1, grpr, elovl7, slc4a1, krt4, lhx9, hmx3, igfbp4, kcnh4a* |
| *areb6* | 42 | 0.019893 | *hcrt, ptgs2, star, f2rl1, fam46a, ttn, pnp, pcsk2, hspa1l, mcoln3, crb1, grpr,*  *creb3l1, elovl7, krt4, npffr1, lhx9, c16orf45, scg2, soat2, tsen54, nos1,*  *rfx4, hpcal4, trpc7, ntng1, ptprn, cyb561, epha2, dennd1b, npvf, sgsm1,*  *wscd1, pde2a, adra1a, vgll2, c2cd4a, hmx3, kcnh4a, ugp2, igfbp4, nr5a1* |
| *zid* | 27 | 0.021993 | *hcrt, ptgs2, ttn, hspa1l, pcsk2, elovl7, slc4a1, krt4, lhx9, tsen54, nos1,*  *rfx4, hpcal4, trpc7, ntng1, myh4, cacng4, ptprn, mmp13, cyb561,*  *dennd1b, pde2a, c2cd4a, hmx3, igfbp4, kcnh4a, nr5a1* |
| *pbx1* | 31 | 0.024504 | *star, ptgs2, ttn, fam46a, pcsk2, crb1, grpr, npffr1, lhx9, scg2, soat2,*  *rfx4, syt10, hpcal4, trpc7, ntng1, cacng4, myh4, cyb561, dennd1b,*  *npvf, sgsm1, wscd1, pde2a, vgll2, adra1a, hmx3, ugp2, igfbp4, kcnh4a,*  *nr5a1* |
| *lyf1* | 23 | 0.027536 | *hcrt, soat2, tsen54, rfx4, hpcal4, trpc7, ntng1, myh4, cacng4, ttn,*  *epha2, pcsk2, npvf, wscd1, pde2a, crb1, creb3l1, adra1a, c2cd4a,*  *npffr1, lhx9, hmx3, kcnh4a* |
| *pax3* | 25 | 0.027855 | *ttn, fam46a, pnp, hspa1l, pcsk2, crb1, grpr, creb3l1, lhx9, rfx4, trpc7,*  *ntng1, myh4, cacng4, ptprn, cyb561, epha2, dennd1b, pde2a, wscd1,*  *vgll2, ugp2, igfbp4, hmx3, nr5a1* |
| *srebp1* | 32 | 0.029786 | *hcrt, star, ptgs2, ttn, fam46a, pnp, hspa1l, pcsk2, crb1, grpr, creb3l1,*  *npffr1, slc4a1, krt4, lhx9, tsen54, nos1, rfx4, ntng1, cacng4, ptprn,*  *cyb561, dennd1b, npvf, sgsm1, wscd1, pde2a, adra1a, ugp2, igfbp4,*  *kcnh4a, nr5a1* |
| *coup* | 24 | 0.030724 | *hcrt, tsen54, nos1, rfx4, hpcal4, ntng1, myh4, ttn, fam46a, cyb561,*  *dennd1b, wscd1, pde2a, grpr, creb3l1, vgll2, adra1a, elovl7, hmx3,*  *ugp2, igfbp4, kcnh4a, c16orf45, nr5a1* |
| *myod* | 30 | 0.033161 | *hcrt, ttn, hspa1l, pcsk2, crb1, grpr, creb3l1, elovl7, slc4a1, lhx9, scg2,*  *soat2, tsen54, nos1, rfx4, hpcal4, ntng1, cacng4, myh4, ptprn, cyb561,*  *epha2, dennd1b, sgsm1, wscd1, pde2a, vgll2, hmx3, kcnh4a, nr5a1* |
| *tal1 beta i tf2* | 29 | 0.035057 | *hcrt, ptgs2, ttn, fam46a, mcoln3, crb1, grpr, creb3l1, c16orf45, scg2,*  *soat2, rfx4, syt10, hpcal4, trpc7, ntng1, myh4, ptprn, cyb561, epha2,*  *dennd1b, sgsm1, pde2a, adra1a, c2cd4a, ugp2, igfbp4, kcnh4a, nr5a1* |
| *nf kappa b* | 21 | 0.036229 | *tsen54, nos1, rfx4, star, ptgs2, syt10, hpcal4, trpc7, ntng1, ttn, fam46a,*  *cyb561, epha2, dennd1b, pcsk2, pde2a, grpr, creb3l1, ugp2, igfbp4, kcnh4a* |
| *cmyb* | 24 | 0.039345 | *hcrt, tsen54, rfx4, star, hpcal4, trpc7, ntng1, myh4, cacng4, ptprn, ttn,*  *cyb561, dennd1b, pcsk2, hspa1l, wscd1, crb1, grpr, creb3l1, slc4a1,*  *npffr1, hmx3, kcnh4a, nr5a1* |
| *crebp1* | 26 | 0.039767 | *ptgs2, ttn, fam46a, pnp, pcsk2, crb1, creb3l1, slc4a1, scg2, nos1, rfx4,*  *syt10, trpc7, ntng1, myh4, cacng4, ptprn, epha2, dennd1b, npvf, sgsm1,*  *pde2a, wscd1, hmx3, igfbp4, nr5a1* |
| *nfkb* | 27 | 0.042004 | *hcrt, star, ptgs2, ttn, fam46a, pcsk2, crb1, grpr, creb3l1, elovl7, lhx9,*  *soat2, tsen54, nos1, rfx4, syt10, ntng1, cacng4, cyb561, epha2, dennd1b,*  *npvf, pde2a, hmx3, igfbp4, kcnh4a, nr5a1* |
| *myb* | 25 | 0.043756 | *hcrt, star, ttn, hspa1l, pcsk2, crb1, slc4a1, lhx9, tsen54, nos1, rfx4,*  *hpcal4, ntng1, myh4, cacng4, cyb561, epha2, dennd1b, sgsm1, wscd1,*  *vgll2, adra1a, kcnh4a, hmx3, nr5a1* |
| *atf* | 19 | 0.045287 | *nos1, rfx4, ntng1, myh4, ptprn, ttn, pnp, epha2, pcsk2, hspa1l, sgsm1,*  *pde2a, wscd1, creb3l1, c2cd4a, slc4a1, hmx3, nr5a1, scg2* |
| *mef2* | 37 | 0.045792 | *hcrt, star, ttn, fam46a, pnp, pcsk2, mcoln3, crb1, grpr, creb3l1, elovl7,*  *npffr1, lhx9, c16orf45, scg2, nos1, rfx4, syt10, hpcal4, trpc7, ntng1,*  *cacng4, myh4, cyb561, epha2, dennd1b, npvf, sgsm1, wscd1, pde2a,*  *adra1a, vgll2, c2cd4a, hmx3, ugp2, igfbp4, kcnh4a* |
| *bach1* | 27 | 0.04716 | *ttn, pnp, pcsk2, mcoln3, grpr, creb3l1, slc4a1, npffr1, krt4, c16orf45,*  *scg2, tsen54, nos1, rfx4, trpc7, ntng1, myh4, cacng4, ptprn, cyb561,*  *mmp13, epha2, dennd1b, npvf, pde2a, wscd1, hmx3* |
| *hnf3b* | 23 | 0.047464 | *rfx4, syt10, trpc7, ntng1, ttn, fam46a, cyb561, dennd1b, pcsk2, npvf,*  *mcoln3, wscd1, pde2a, crb1, grpr, creb3l1, vgll2, adra1a, krt4, lhx9,*  *ugp2, c16orf45, scg2* |
| *roaz* | 25 | 0.048245 | *ptgs2, ttn, hspa1l, pcsk2, crb1, creb3l1, krt4, lhx9, c16orf45, tsen54,*  *nos1, rfx4, trpc7, ntng1, myh4, cacng4, ptprn, epha2, sgsm1, pde2a,*  *wscd1, adra1a, kcnh4a, hmx3, nr5a1* |
| *ap1fj* | 18 | 0.048759 | *soat2, hpcal4, trpc7, f2rl1, myh4, ptprn, ttn, fam46a, epha2, dennd1b,*  *pcsk2, hspa1l, sgsm1, pde2a, elovl7, krt4, c16orf45, nr5a1* |
| *nkx61* | 24 | 0.049298 | *tsen54, nos1, rfx4, syt10, f2rl1, ntng1, cacng4, ttn, cyb561, dennd1b,*  *pcsk2, npvf, sgsm1, wscd1, crb1, creb3l1, adra1a, elovl7, lhx9, hmx3,*  *ugp2, c16orf45, scg2, nr5a1* |
| *cdp* | 31 | 0.05143 | *hcrt, star, ptgs2, ttn, fam46a, hspa1l, pcsk2, crb1, grpr, creb3l1, elovl7,*  *npffr1, slc4a1, lhx9, scg2, rfx4, syt10, trpc7, ntng1, cacng4, myh4, ptprn,*  *mmp13, cyb561, dennd1b, npvf, wscd1, pde2a, adra1a, hmx3, nr5a1* |
| *hoxa3* | 23 | 0.053052 | *hcrt, tsen54, nos1, rfx4, star, ntng1, myh4, ptprn, ttn, fam46a, pnp,*  *cyb561, epha2, dennd1b, pcsk2, hspa1l, npvf, pde2a, elovl7, lhx9,*  *hmx3, kcnh4a, nr5a1* |
| *foxo1* | 23 | 0.053052 | *hcrt, soat2, tsen54, rfx4, ptgs2, ntng1, ttn, fam46a, pnp, cyb561,*  *dennd1b, pcsk2, npvf, wscd1, pde2a, crb1, grpr, lhx9, hmx3, igfbp4,*  *kcnh4a, c16orf45, nr5a1* |
| *aml1* | 38 | 0.05512 | *hcrt, f2rl1, fam46a, ttn, pnp, pcsk2, crb1, grpr, creb3l1, krt4, npffr1,*  *lhx9, c16orf45, soat2, tsen54, rfx4, syt10, hpcal4, trpc7, ntng1, cacng4,*  *myh4, ptprn, mmp13, cyb561, epha2, dennd1b, npvf, sgsm1, wscd1,*  *pde2a, adra1a, vgll2, c2cd4a, kcnh4a, ugp2, igfbp4, nr5a1* |
| *er* | 27 | 0.056868 | *hcrt, f2rl1, ttn, hspa1l, pcsk2, crb1, slc4a1, npffr1, soat2, tsen54, nos1,*  *rfx4, trpc7, ntng1, cacng4, myh4, ptprn, epha2, npvf, sgsm1, pde2a,*  *vgll2, adra1a, c2cd4a, hmx3, kcnh4a, nr5a1* |
| *srf* | 35 | 0.062313 | *hcrt, ptgs2, star, ttn, fam46a, pcsk2, crb1, grpr, creb3l1, elovl7, npffr1,*  *slc4a1, krt4, lhx9, soat2, tsen54, rfx4, hpcal4, trpc7, ntng1, cacng4,*  *myh4, ptprn, cyb561, dennd1b, npvf, sgsm1, pde2a, adra1a, vgll2,*  *hmx3, ugp2, igfbp4, kcnh4a, nr5a1* |
| *stat1* | 22 | 0.062463 | *soat2, tsen54, nos1, rfx4, trpc7, ntng1, myh4, cacng4, ptprn, ttn, pnp,*  *epha2, pcsk2, sgsm1, pde2a, grpr, creb3l1, adra1a, lhx9, hmx3,*  *c16orf45, scg2* |
| *ik3* | 24 | 0.064382 | *hcrt, soat2, rfx4, star, trpc7, ntng1, myh4, ttn, mmp13, pnp, cyb561,*  *epha2, pcsk2, wscd1, pde2a, crb1, creb3l1, vgll2, elovl7, lhx9, hmx3,*  *igfbp4, kcnh4a, nr5a1* |
| *hen1* | 30 | 0.065283 | *hcrt, ttn, pnp, hspa1l, pcsk2, crb1, creb3l1, npffr1, slc4a1, scg2, soat2,*  *tsen54, nos1, rfx4, hpcal4, trpc7, ntng1, cacng4, myh4, ptprn, cyb561,*  *epha2, sgsm1, pde2a, vgll2, adra1a, hmx3, igfbp4, kcnh4a, nr5a1* |
| *nkx25* | 32 | 0.067464 | *star, ttn, fam46a, pnp, pcsk2, mcoln3, crb1, grpr, creb3l1, elovl7,*  *npffr1, slc4a1, lhx9, c16orf45, scg2, rfx4, syt10, trpc7, ntng1,*  *cacng4, myh4, cyb561, epha2, dennd1b, npvf, sgsm1, wscd1,*  *pde2a, adra1a, hmx3, ugp2, igfbp4* |
| *ik1* | 20 | 0.06812 | *hcrt, tsen54, rfx4, hpcal4, trpc7, myh4, ttn, mmp13, cyb561, dennd1b,*  *pcsk2, pde2a, crb1, creb3l1, adra1a, elovl7, slc4a1, hmx3, kcnh4a, ugp2* |
| *ap2 rep* | 24 | 0.069976 | *hcrt, tsen54, rfx4, hpcal4, trpc7, ntng1, ptprn, ttn, pnp, cyb561, epha2,*  *pcsk2, hspa1l, sgsm1, wscd1, pde2a, crb1, creb3l1, adra1a, npffr1,*  *lhx9, ugp2, kcnh4a, nr5a1* |
| *pax6* | 28 | 0.070385 | *star, ptgs2, ttn, fam46a, hspa1l, pcsk2, crb1, grpr, elovl7, npffr1, lhx9,*  *c16orf45, soat2, tsen54, nos1, rfx4, syt10, trpc7, ntng1, cacng4,*  *cyb561, dennd1b, pde2a, wscd1, c2cd4a, hmx3, igfbp4, nr5a1* |
| *freac3* | 23 | 0.074707 | *hcrt, rfx4, trpc7, f2rl1, ntng1, myh4, ptprn, ttn, pnp, cyb561, dennd1b,*  *pcsk2, npvf, wscd1, pde2a, crb1, grpr, creb3l1, adra1a, elovl7, lhx9,*  *nr5a1, scg2* |
| *comp1* | 26 | 0.074803 | *star, ptgs2, ttn, fam46a, pnp, hspa1l, pcsk2, creb3l1, elovl7, slc4a1,*  *krt4, c16orf45, soat2, tsen54, trpc7, cacng4, myh4, cyb561, sgsm1,*  *pde2a, wscd1, vgll2, adra1a, igfbp4, kcnh4a, nr5a1* |
| *cart1* | 25 | 0.075488 | *ttn, fam46a, hspa1l, pcsk2, crb1, grpr, creb3l1, elovl7, lhx9, scg2,*  *nos1, rfx4, trpc7, ntng1, myh4, cacng4, cyb561, mmp13, dennd1b,*  *npvf, pde2a, vgll2, ugp2, igfbp4, nr5a1* |
| *arp1* | 25 | 0.077058 | *hcrt, ttn, hspa1l, crb1, creb3l1, elovl7, slc4a1, lhx9, tsen54, nos1,*  *rfx4, hpcal4, trpc7, ptprn, epha2, dennd1b, npvf, sgsm1, pde2a,*  *wscd1, adra1a, igfbp4, kcnh4a, hmx3, nr5a1* |
| *olf1* | 24 | 0.079904 | *hcrt, soat2, tsen54, nos1, rfx4, hpcal4, cacng4, ptprn, ttn, pnp,*  *cyb561, epha2, dennd1b, pcsk2, hspa1l, sgsm1, wscd1, pde2a,*  *creb3l1, adra1a, vgll2, c2cd4a, hmx3, kcnh4a* |
| *tal1 alpha e47* | 13 | 0.080153 | *rfx4, hpcal4, ntng1, cacng4, fam46a, ttn, cyb561, pcsk2, sgsm1,*  *pde2a, crb1, adra1a, c2cd4a* |
| *hfh3* | 22 | 0.080213 | *hcrt, tsen54, nos1, star, trpc7, ntng1, myh4, ttn, cyb561, epha2,*  *dennd1b, pcsk2, wscd1, pde2a, grpr, adra1a, c2cd4a, elovl7, npffr1,*  *lhx9, hmx3, ugp2* |
| *brn2* | 26 | 0.081655 | *hcrt, ttn, fam46a, pcsk2, crb1, grpr, elovl7, npffr1, lhx9, c16orf45,*  *scg2, nos1, rfx4, ntng1, myh4, epha2, dennd1b, npvf, wscd1, vgll2,*  *adra1a, c2cd4a, hmx3, igfbp4, ugp2, kcnh4a* |
| *tcf11 mafg* | 27 | 0.084076 | *hcrt, ttn, pcsk2, mcoln3, crb1, slc4a1, krt4, lhx9, c16orf45, tsen54,*  *nos1, rfx4, hpcal4, trpc7, ntng1, myh4, ptprn, cyb561, mmp13,*  *epha2, dennd1b, sgsm1, pde2a, wscd1, hmx3, kcnh4a, nr5a1* |
| *bach2* | 23 | 0.084227 | *soat2, rfx4, hpcal4, trpc7, ntng1, ptprn, ttn, mmp13, pnp, cyb561,*  *epha2, dennd1b, pcsk2, npvf, mcoln3, pde2a, grpr, creb3l1,*  *adra1a, slc4a1, npffr1, igfbp4, c16orf45* |
| *evi1* | 39 | 0.085777 | *hcrt, ptgs2, star, f2rl1, fam46a, ttn, pnp, hspa1l, pcsk2, mcoln3,*  *crb1, grpr, creb3l1, elovl7, slc4a1, lhx9, c16orf45, scg2, soat2,*  *nos1, rfx4, syt10, trpc7, ntng1, cacng4, myh4, cyb561, dennd1b,*  *npvf, sgsm1, wscd1, pde2a, adra1a, vgll2, hmx3, ugp2, igfbp4,*  *kcnh4a, nr5a1* |
| *e4bp4* | 23 | 0.086443 | *nos1, rfx4, ptgs2, syt10, ntng1, myh4, cacng4, ttn, fam46a,*  *dennd1b, pcsk2, npvf, pde2a, crb1, creb3l1, vgll2, adra1a, elovl7,*  *lhx9, hmx3, ugp2, scg2, nr5a1* |
| *cebp* | 37 | 0.086476 | *hcrt, star, fam46a, ttn, pcsk2, crb1, grpr, creb3l1, elovl7, npffr1,*  *krt4, lhx9, scg2, soat2, tsen54, nos1, rfx4, syt10, trpc7, ntng1,*  *cacng4, myh4, ptprn, cyb561, epha2, dennd1b, npvf, wscd1,*  *pde2a, adra1a, vgll2, c2cd4a, hmx3, ugp2, igfbp4, kcnh4a, nr5a1* |
| *foxd3* | 19 | 0.086687 | *nos1, rfx4, syt10, hpcal4, trpc7, ntng1, ttn, fam46a, cyb561,*  *dennd1b, pcsk2, npvf, pde2a, crb1, adra1a, lhx9, igfbp4,*  *scg2, nr5a1* |
| *spz1* | 18 | 0.089753 | *tsen54, rfx4, hpcal4, ntng1, myh4, ptprn, pnp, cyb561, epha2,*  *pcsk2, pde2a, wscd1, creb3l1, slc4a1, krt4, hmx3, kcnh4a, ugp2* |
| *gata6* | 11 | 0.094616 | *pcsk2, star, pde2a, grpr, ntng1, creb3l1, ttn, pnp, c16orf45,*  *dennd1b, nr5a1* |
| *oct* | 24 | 0.098239 | *tsen54, nos1, rfx4, syt10, trpc7, f2rl1, ntng1, myh4, cacng4, ttn,*  *cyb561, dennd1b, pcsk2, npvf, wscd1, pde2a, crb1, adra1a,*  *c2cd4a, elovl7, lhx9, hmx3, ugp2, kcnh4a* |
| *freac2* | 18 | 0.099043 | *tsen54, nos1, ntng1, myh4, cacng4, ttn, mmp13, cyb561, dennd1b,*  *pcsk2, sgsm1, crb1, creb3l1, vgll2, adra1a, slc4a1, ugp2, nr5a1* |
| *msx1* | 22 | 0.099727 | *soat2, rfx4, trpc7, f2rl1, ntng1, cacng4, ttn, pnp, cyb561, dennd1b,*  *npvf, mcoln3, pde2a, grpr, adra1a, c2cd4a, elovl7, lhx9, hmx3, ugp2,*  *kcnh4a, nr5a1* |
